# Complementary volume electron microscopy-based approaches reveal ultrastructural changes in germline intercellular bridges

**DOI:** 10.1242/jcs.264713

**Published:** 2026-05-26

**Authors:** Irina Kolotuev, Sophie Khazanov, Abigayle Williams, Caroline Kizilyaprak, Stephanie Pellegrino, Lindsay Lewellyn

**Affiliations:** ^1^ University of Lausanne, Faculté de biologie et de médecine, Electron Microscopy Facility, CH-1015 Lausanne, Switzerland; ^2^ University of Lausanne, Faculté de biologie et de médecine, Department of Biological Sciences, CH-1005 Lausanne, Switzerland; ^3^ Medical Laboratory Sciences Department, Jerusalem Multidisciplinary College, Jerusalem 91010, Israel; ^4^ Butler University, Department of Biological Sciences, Indianapolis, IN 46208, USA

**Keywords:** Volume electron microscopy, *Drosophila melanogaster*, Ring canal, Egg chamber, Intercellular bridge

## Abstract

The *Drosophila melanogaster* egg chamber is a powerful model system to study germline intercellular bridges, or ring canals, which connect the developing oocyte to supporting nurse cells. Despite their importance, it is technically difficult to use electron microscopy-based approaches to monitor changes in ring canal structure. Here, we utilize a complementary set of volume electron microscopy-based approaches to visualize ultrastructural changes in the germline ring canals. The combination of array tomography and focused ion beam scanning electron microscopy provided insight into previously unappreciated aspects of ring canal structure. We quantified differences in ring canal size and thickness within and between germline cell clusters and visualized the formation of membrane interdigitations near the ring canals much earlier than previously reported. Reconstruction of multiple egg chambers provided insight into the three-dimensional orientation of these extensive cell–cell contacts. Finally, we identified a novel membrane structure that appeared to line the interior of the ring canal lumen. This imaging framework could be applied to other tissues with technical challenges, in which the small structure of interest is located within a large sample volume.

## Teaser

**Highlighted Article:** FIB-SEM and AT-SEM reveal inter- and intra-cluster ultrastructural differences in germline ring canals, and the early appearance and orientation of extensive membrane interdigitations in the *Drosophila* egg chamber.

## INTRODUCTION

Intercellular bridges are essential structures that connect somatic cells to each other or developing sperm and eggs to other gametes or to supporting ‘nurse’ cells. Germline intercellular bridges have been observed throughout the animal kingdom from insects to humans; they allow the sharing of haploid gene products, synchronization of meiotic entry, and delivery of materials to support patterning and early embryonic development (Spradling et al., 2022). Although these structures are essential for fertility in many organisms, there is still much to learn about how intercellular bridges are formed, how they are structurally stabilized, and how they expand.

**Fig. 1.**
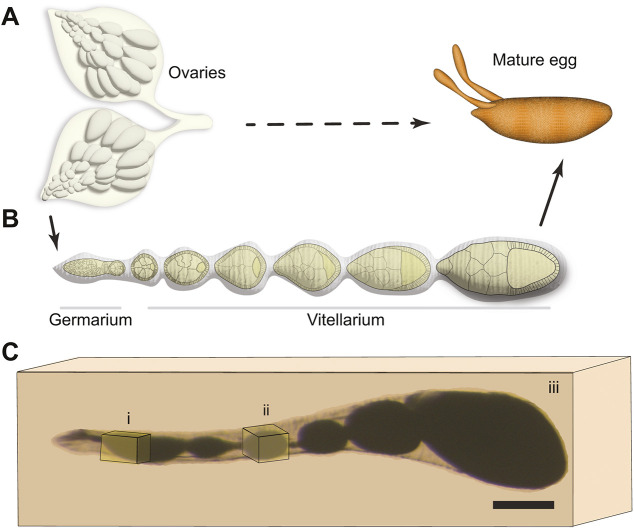
**Overview of oogenesis in *Drosophila melanogaster*.** (A,B) Schematic representation of the organization of the ovarioles within the ovaries. Diagram of a single ovariole showing different stages (germarium through stage 10) of oogenesis before forming a mature egg. (C) Image of an ovariole processed for electron microscopy (EM) and embedded in epoxy resin. Boxes i and ii, indicate the approximate region of the ovariole that was covered by each of the two focused ion beam (FIB)-scanning EM (SEM) datasets (Movies 1a,b at doi:10.6084/m9.figshare.c.8450008) that are described in subsequent figures. The large box enclosing the entire ovariole (iii) represents the sample covered using array tomography (AT)-SEM. Scale bar: 100 μm.

The largest and most well-studied intercellular bridges connect germline cells within the developing egg chamber of the fruit fly *Drosophila melanogaster*. The egg chambers, which ultimately give rise to the mature fly egg, are formed within a structure called the germarium, which lies at the anterior end of the ovariole. Over a dozen ovarioles are found within each ovary; these ovarioles contain a series of egg chambers at different stages of development (Fig. 1A,B). Egg chamber formation begins with the division of a germline stem cell; one daughter cell, the cystoblast, divides mitotically to generate the sixteen germline cells. These divisions are coordinated across the cluster by a membrane–cytoskeletal structure, the fusome; unequal inheritance of the fusome establishes one of these sixteen germline cells as the oocyte, and the remaining cells serve as supporting nurse cells (de Cuevas and Spradling, 1998; Deng and Lin, 1997; Diegmiller et al., 2023; Grieder et al., 2000; Lighthouse et al., 2008; Lin et al., 1994; Lin and Spradling, 1995; Nashchekin et al., 2021; Snapp et al., 2004). Division of the germline cells is followed by incomplete cytokinesis and stabilization of an intercellular bridge, called the ring canal, which connects the germline cells within the cluster (Ong and Tan, 2010; Robinson et al., 1994; Spradling et al., 2022). Once formed, the ring canals must expand ∼10-fold in diameter, allowing materials to pass from the nurse cells to the oocyte, which is essential to support the production of a viable mature egg.

The germline ring canals in *Drosophila* are significantly larger and undergo more extensive growth than intercellular bridges that have been studied in other organisms. The requirement for structural stability during regulated growth is likely related to the multi-layered structure of the ring canals, which is more complex than the single ‘rim’ that has been reported for other intercellular bridges (Haglund et al., 2011). The germline ring canals consist of what is defined as an ‘inner rim’ of actin and actin-binding proteins and a thickened region of the nurse cell membrane, which has been termed the ‘outer rim’. In addition, extensive membrane invaginations or interdigitations have been observed, at least at the later stages of oogenesis (Loyer et al., 2015; Tilney et al., 1996), but the composition of this region and its role during oogenesis have not been determined. Fluorescence microscopy-based studies have revealed much about ring canal formation and growth (Gerdes et al., 2020; Hudson et al., 2019; Price et al., 2023; Robinson et al., 1997; Sokol and Cooley, 1999; Tilney et al., 1996; Xue and Cooley, 1993); however, the resolution of light microscopy is insufficient to visualize nanoscale structures. Moreover, because fluorescence microscopy involves the labeling of specific proteins, there are likely other aspects of the developing germline cells that are being overlooked.

Electron microscopy (EM) provides the resolution and cellular context necessary to better understand these essential germline cell structures. Transmission EM (TEM) is often considered the gold standard of EM, providing angstrom-level resolution and access to the entire morphome of the cell or tissue. Studies using TEM have revealed aspects of germline cell and ring canal structure, such as the presence of the membrane interdigitations surrounding the ring canals and the changes in actin filament number and length within the inner rim (Loyer et al., 2015; Mahowald, 1971; Riparbelli et al., 2022; Tilney et al., 1996), which would be undetectable by light microscopy. Although a powerful tool, TEM is limited in the sample area and volume that can be imaged and in the electron beam penetration (Fig. S1A,B), and it can be challenging to identify or analyze structures of interest without clearly understanding their spatial orientation.

The desire to collect three-dimensional (3D) ultrastructural data from tissues and organs has catalyzed a revolution in the EM field, focused on promoting the use and accessibility of volume EM (vEM) approaches (Collinson et al., 2023). vEM includes several powerful methods, many of which use scanning EM (SEM), to obtain high-resolution images through a large sample volume. Focused ion beam (FIB), scanning block face and array tomography (AT) primarily differ in the way the sample surface is exposed for subsequent imaging, ranging between automatic and manual processing (Fig. S1A,B) (Collinson et al., 2023; Peddie et al., 2022). Unfortunately, some experimental questions cannot be completely answered by a single vEM approach; these instead require a carefully selected combination of vEM techniques.

Our desire to understand how the ring canals maintain a stable connection between germline cells while also undergoing significant growth motivated us to explore the best EM approaches to monitor ring canal development during oogenesis. After considering the strengths and limitations of different vEM approaches, we have selected a combination of FIB-SEM and AT-SEM to visualize the germline ring canals in the developing egg chamber. This combination has provided significant insight into the ultrastructural changes that occur during oogenesis. We were able to quantify differences in ring canal size within and between germline cell clusters at different developmental stages. Within a cluster, ring canal size correlates with lineage; the largest ring canals were formed during the first division, and the smallest ring canals were formed during the fourth mitotic division. We observed the formation of membrane interdigitations in the vicinity of ring canals much earlier than previously reported, and careful reconstruction of ring canals from multiple egg chambers provided novel insight into the abundance and orientation of these extensive cell–cell contacts. These high-content datasets also revealed a novel membrane structure that appeared to line the interior of the ring canal lumen. In addition to our findings, this work can serve as an example to guide other researchers who wish to use vEM to study the same or similar tissue types. These datasets can be further mined by researchers studying a variety of topics in oogenesis.

## RESULTS

### Analysis of the germline ring canals requires volumetric, high-resolution imaging

**Fig. 2.**
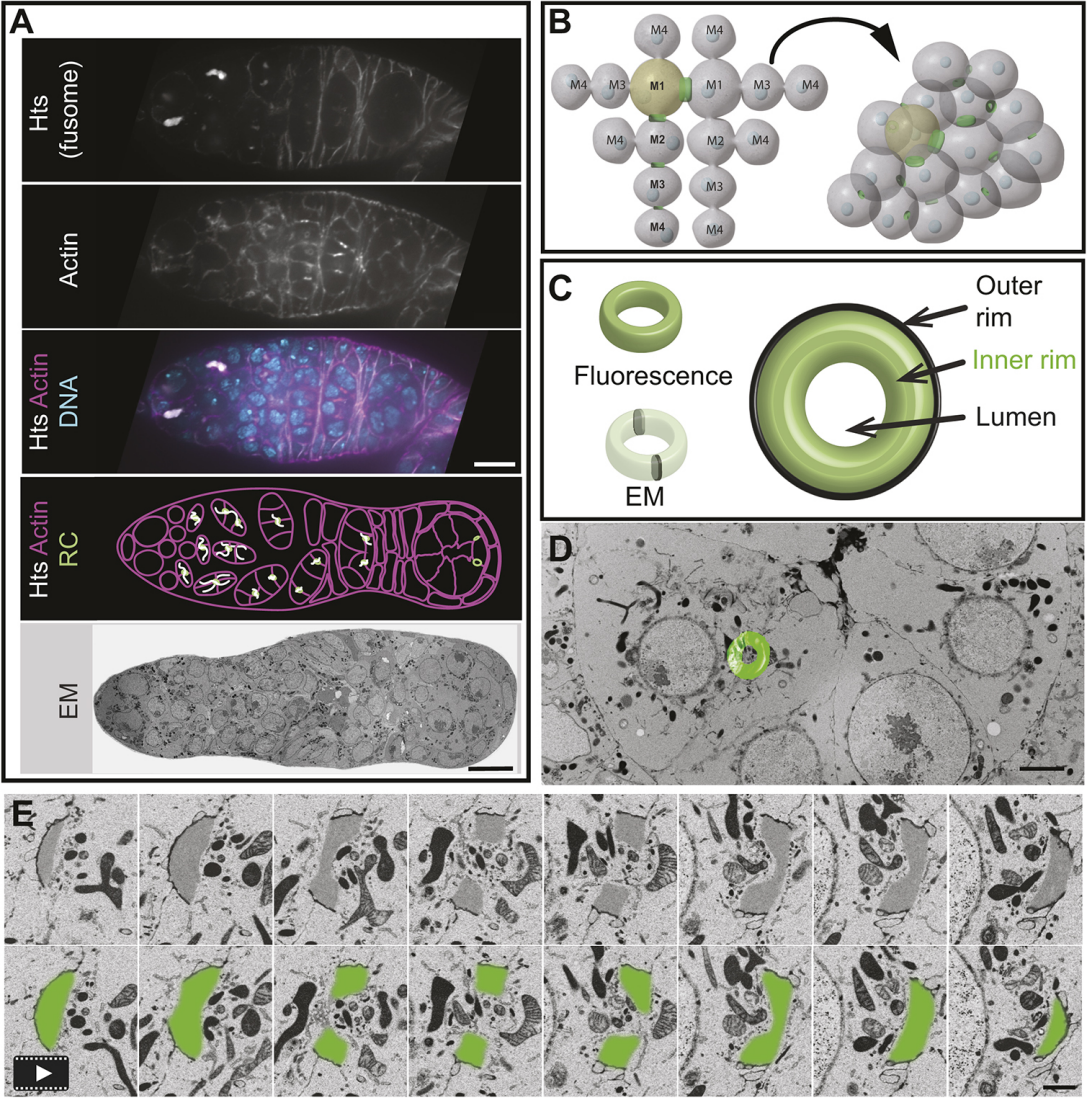
***Drosophila* egg chamber development and germline ring canals.** (A) The first three panels show single-section confocal fluorescence images of the germarium stained with an anti-Hts antibody to label the fusome (white), phalloidin to label actin (magenta) and DAPI (cyan). Scale bar: 10 μm. The fourth panel depicts a cartoon showing the localization of Hts, actin and the ring canals (RCs) in the germarium. The last panel is an image of a single section through the germarium of the AT-SEM image, which shows not only the richness of an EM dataset, but also highlights the complexity of the structure. Scale bar: 10 μm. (B) The diagram shows the stereotypical organization of the germline cell cluster as it often appears in drawings (left) compared to the manner in which it is packaged within the tissue (right). M1, M2, M3 and M4 indicate the first, second, third and fourth mitotic divisions. (C) Diagram showing the structure of the ring canal and how it might be observed by fluorescence microscopy or a single EM section. (D,E) Serial sectioning through the same ring canal results in different sectioning orientations and a highly variable appearance. Therefore, volume EM (vEM; shown in green overlay in D) provides the most informative view of these structures. FIB-SEM images of different sections through the same ring canal (from a stage 4 egg chamber) (E) demonstrate the different ways that the same ring canal can appear. The inner rim of the ring canal in every other section has been pseudocolored in the bottom row. Scale bars: 3 μm (D); 1 μm (E).

Egg chamber formation begins at the anterior end of the germarium, where a germline stem cell divides to produce a daughter cystoblast. This cystoblast subsequently undergoes four synchronized mitotic divisions, producing a cluster of 16 germline cells. These cell divisions are incomplete, and a stable intercellular bridge, or ring canal, will connect the daughter cells. Once formed, the compact germline cell clusters (Fig. 2A,B) advance in their development as they move away from the stem cell niche. Mature ring canals exhibit a distinct multi-layered architecture, consisting of an electron-dense outer region and an inner rim of lower electron density composed primarily of actin filaments and associated actin-binding proteins (Fig. 2C). As oogenesis proceeds, the overall size of the egg chamber increases, making it more difficult to locate and image the entire ring canal within older egg chambers. Furthermore, because ring canals are rarely oriented horizontally to the sectioning plane, it is critical to collect many sections through the ring canals to generate a 3D model (Fig. 2D,E; Fig. S1C). For our analysis, we decided on a combination of two vEM approaches, FIB-SEM and AT-SEM, to visualize ring canal development throughout oogenesis. FIB-SEM provided us with a high-resolution, well-aligned dataset that covered multiple young germline clusters, whereas AT-SEM allowed us to efficiently identify sections that contained germline ring canals from a much larger sample volume; these targeted regions were imaged at high resolution (Fig. 1C; Fig. S1A,B).

### FIB-SEM reveals early ultrastructural differences in ring canal size and thickness within germline cell clusters

Because the germline cells divide in a stereotypical pattern, the lineage of each germline cell and connecting ring canal can be assigned. For example, a single ring canal is formed after the first mitotic division (M1), two are formed after the second mitotic division (M2), four are formed after the third mitotic division (M3), and eight are produced by the final mitotic division (M4) (Fig. 2B). Only a few studies have described lineage-based differences in ring canal size or structure. One TEM-based study reported that within the germarium, ring canal diameter and accumulation of material in the inner rim correlated with lineage (Koch and King, 1969); however, a detailed analysis of this ultrastructural variation was not performed. More recent light microscopy-based studies reported lineage-based differences in the degree of contractile ring constriction and midbody size; with each germline cell division, the contractile ring constricted further and produced a smaller midbody, such that the ring canals that formed from the first mitotic division had a larger starting diameter compared to those formed following the subsequent divisions (Ong and Tan, 2010; Price et al., 2023). Recent work has demonstrated that there are lineage-based differences in ring canal scaling. Specifically, the largest M1 ring canals increase in size more slowly than the smaller ring canals derived from later mitotic divisions (Shaikh et al., 2024). Our high-resolution FIB-SEM datasets provided us with the opportunity to further explore the lineage-based ultrastructural differences and develop testable hypotheses to explain the observed differences in ring canal scaling.

**Fig. 3.**
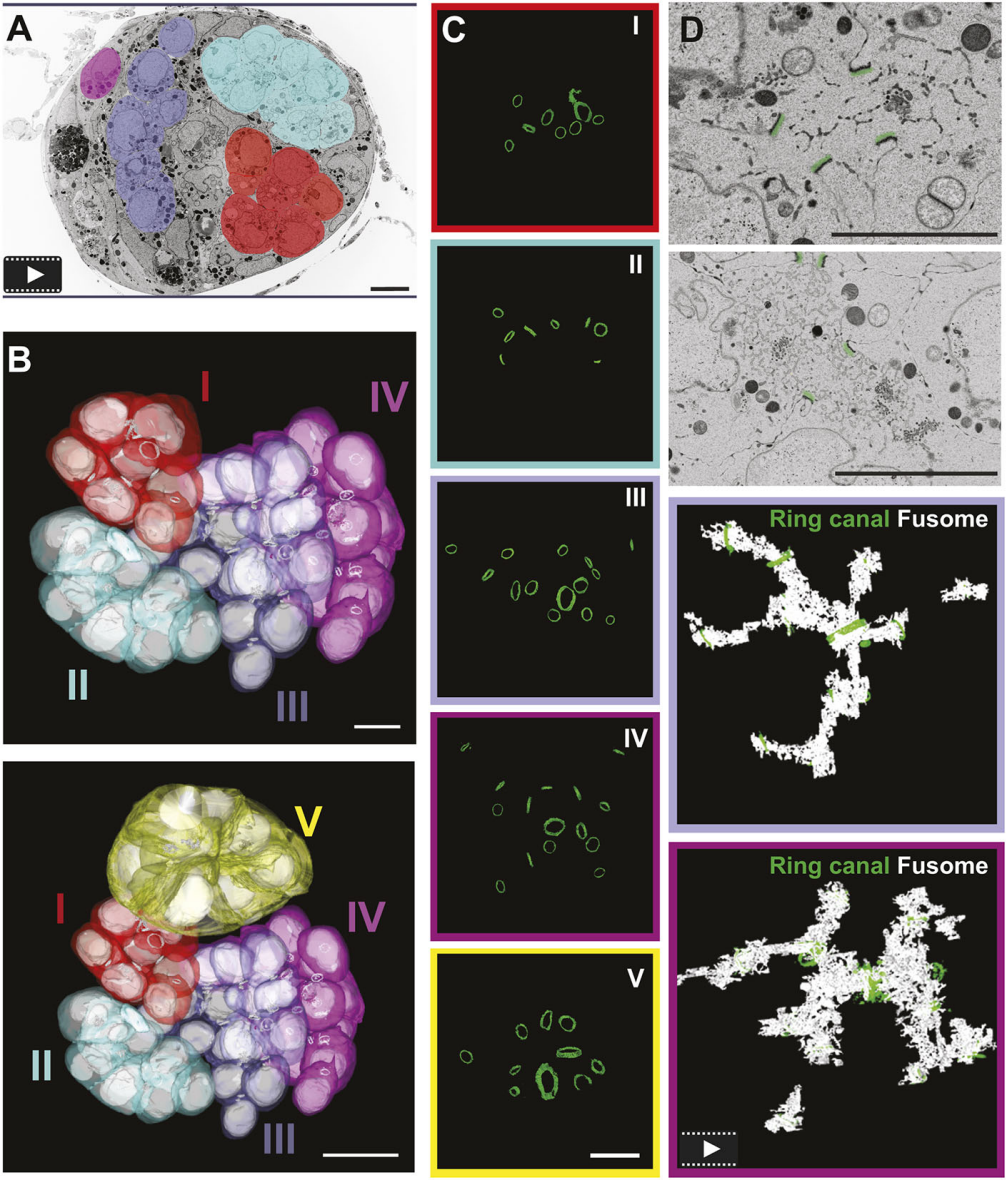
**FIB-SEM analysis of multiple germline cell clusters within the germarium reveals differences in ring canal size and structure.** FIB-SEM was used to acquire 10.2 nm resolution images of part of the germarium. (A) A single section from the FIB-SEM stack shows the content-rich images; germline cells are pseudocolored to indicate the germline cell cluster. Scale bar: 5 μm. (B) Rendering of the germline cell membranes, ring canals and nuclei within all clusters contained in the stack. Scale bars: 5 μm (top); 10 μm (bottom). (C) The isosurface tool was used to render the ring canals from all clusters shown in B; it captures material passing through the ring canals, which was manually removed in these images for clarity. These 3D models allowed us to visualize the differences in ring canal size, thickness and spatial orientation within each cluster. Scale bar: 5 μm. (D) Single sections from the FIB-SEM image stack showing cross sections of the fusome passing through multiple ring canals (pseudocolored in green). Rendering using the isosurface tool generates a model of the branched fusome structure in two germline clusters (III and IV) from region 2b of the germarium. Scale bars: 5 μm.

Due to the compact nature of the germarium, we expected that a single FIB-SEM dataset could contain many germline cell clusters (Fig. 1C, box I; Movie 1a at doi:10.6084/m9.figshare.c.8450008), allowing us to compare ring canal size and toroid thickness within and between different germline cell clusters. The first FIB-SEM dataset was acquired at a resolution of ∼10 nm in the *xy*-plane and 25 nm in the *z*-plane (Fig. 3A; Movie 3a at doi:10.6084/m9.figshare.c.8450008). To establish the developmental sequence and to understand the tissue ‘landscape’, we used the IMOD program to manually segment the contours of germline cell membranes and the nuclei within these cells (Kremer et al., 1996). We could identify five germline cell clusters at different developmental stages; we color-coded them based on their connections, which allowed us to determine the lineage and position of cells within each cluster (Fig. 3A,B; Movie 3a at doi:10.6084/m9.figshare.c.8450008). Although we do not have complete volumes for all clusters, we were able to conclude that two clusters (I and II) are located within region 2a of the germarium, two clusters are within region 2b (III and IV), and one cluster is in region 3/stage 1 (Fig. 3B) [see Ong and Tan (2010) for a description of germarium regions]. We found that the ring canals within the younger germline cell clusters (I and II) were smaller in diameter and appeared thinner than those in the older clusters (III–V). The average ring canal diameter measured from the darker outer rim was 1.27±0.28 μm (indicated as mean±s.d.) in region 2a (clusters I and II), 1.35±0.32 μm in region 2b (clusters III and IV), and 1.79±0.61 μm in region 3/stage 1 (cluster V). Even though our measurements are precise, they are not absolute as the tissue tends to shrink ∼10% during the dehydration and embedding steps; however, the observed lineage- and stage-specific variations in the ratio are consistent with earlier findings (Mahowald and Strassheim, 1970; Ong and Tan, 2010; Shaikh et al., 2024).

To segment the structurally complex ring canals, we used the IMOD isosurface tool. This nonselective tracking method often results in a less precise tracing of the structure of interest, tracking all structures of a similar threshold within the analyzed area. Therefore, in addition to the toroid components, the material passing through the rings and in their periphery had to be manually removed. Despite this, we could visualize the position, relative size and orientation of all ring canals within the dataset (Fig. 3C; Movie 3a at doi:10.6084/m9.figshare.c.8450008).

While analyzing ring canals in different clusters of the germarium, we could not ignore the presence of the fusome. The fusome is an essential and conserved germline cell structure composed of membrane and cytoskeletal elements, such as the protein Hts (Fig. 2A). Of the sixteen germline cells, two contain four ring canals; evidence suggests that of these two pro-oocytes, the cell that inherits a larger portion of the fusome will become the oocyte, suggesting that the fusome also plays a role in oocyte determination (de Cuevas and Spradling, 1998; Deng and Lin, 1997; Grieder et al., 2000; Lighthouse et al., 2008; Lin et al., 1994; Lin and Spradling, 1995; Nashchekin et al., 2021; Spradling et al., 1997). Despite its importance, very little analysis of the fusome has been done using vEM. Because this structure was obvious in our FIB-SEM dataset, we used many individual isosurface volumes to render the entire fusome from the most well-sampled clusters in the dataset, clusters III and IV (both from region 2b of the germarium). In region 2b, the mitotic divisions have ended, and the fusome undergoes disassembly. In cluster III, the fusome still passed through all visible ring canals (Fig. 3D). In cluster IV, which might be slightly older, we could still see it passing through or near all but one of the fifteen ring canals. Both clusters showed regions of discontinuity within the germline cells that were not directly connected to the pro-oocytes (Fig. 3D; Movie 3b at doi:10.6084/m9.figshare.c.8450008). Additional analysis of this dataset or new FIB-SEM datasets could be performed to fully characterize the assembly and breakdown of this essential germline cell structure.

### FIB-SEM revealed lineage-specific differences in ring canal size and thickness

In addition to the differences in ring canal size between clusters of different developmental stages, our analysis revealed obvious differences in the size and thickness of ring canals within each cluster. It has previously been shown that, at least beginning at stage 3, there is a hierarchy to nurse cell size; the four nurse cells directly connected to the oocyte are larger than the cells that are separated from the oocyte by two ring canals, and so on (Diegmiller et al., 2021; Imran Alsous et al., 2017). One possibility is that ring canal size also varies based on distance from the oocyte; if this were the case, then the ring canals that connect the first layer of nurse cells directly to the oocyte would be the largest, and the ring canals would become progressively smaller the further away they are from the oocyte (Diegmiller et al., 2021; Imran Alsous et al., 2017). Alternatively, ring canal size could be based on lineage. It has been demonstrated that the ring canals that are formed from different mitotic divisions constrict to different endpoints, with the ring canal that originated during the first mitotic division (M1) reaching the largest final diameter and the ring canals that originate from the second, third and fourth mitotic divisions (M2, M3 and M4, respectively) reaching progressively smaller final diameters (Ong and Tan, 2010); the sizes of the midbodies also correlate with lineage (Price et al., 2023). Therefore, we could use vEM to compare ring canal size and thickness based on location and lineage within the germline cell clusters.

**Fig. 4.**
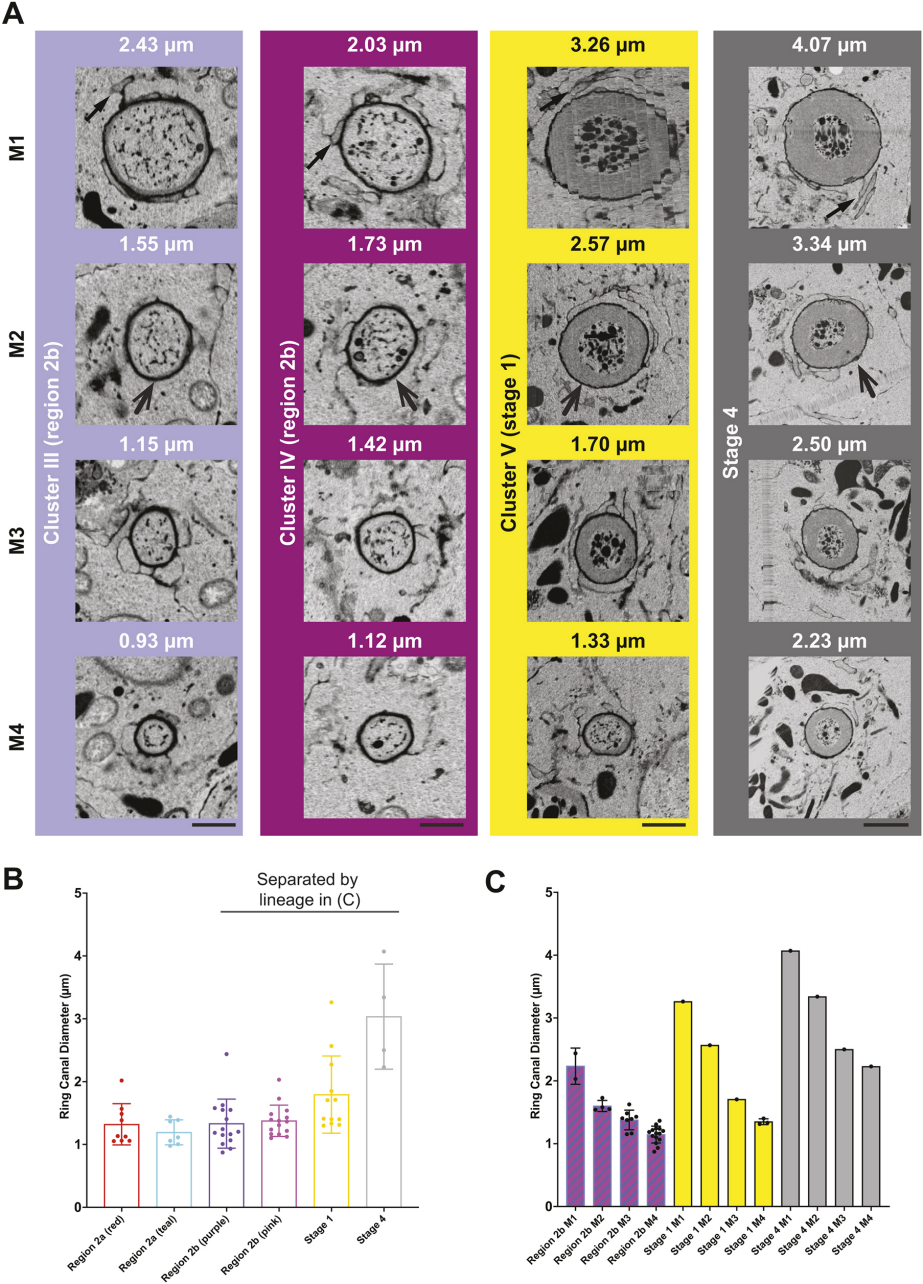
**Ultrastructural differences in ring canals based on stage and lineage.** (A) The Slicer tool was used to generate end-on views of ring canals from each cluster; M1–M4 indicate the mitotic division of origin of each ring canal. Black arrows point to the electron-dense outer region. Measurements above the images indicate ring canal diameter. Scale bars: 1 μm. (B) Average ring canal diameter from each germline cell cluster; individual ring canal measurements are shown, and error bars show standard deviation. *n*=9 (red cluster), 7 (teal cluster), 15 (purple cluster), 15 (pink cluster), 12 (yellow cluster) or 4 (stage 4) ring canals. Only the four posterior ring canals from the stage 4 egg chamber are included. (C) Ring canals with confirmed lineages from region 2b (combined purple and pink clusters; *n*=2 for M1, 4 for M2, 8 for M3 and 15 for M4), stage 1 (*n*=1 for M1, M2 and M3, and *n*=3 for M4) and stage 4 (*n*=1 for each lineage) egg chambers are shown.

To gain further insight into the inter- and intra-cluster differences in ring canal size and thickness, we acquired a FIB-SEM dataset of an older egg chamber (stage 4; Fig. 1C, box ii; Movie 1b at doi:10.6084/m9.figshare.c.8450008) obtained from a deeper level of the same sample. The egg chamber at this stage is larger than the germline clusters within the germarium; owing to the technical limitations of the FIB-SEM microscope, we were unable to capture the entire egg chamber volume. We were, however, able to image portions of all nurse cells, the entire oocyte and eleven ring canals. With these two vEM datasets, we could use the Slicer tool within IMOD to orient all ring canals in the same way and compare ring canal structure. We found that lineage was a more reliable predictor of ring canal size and thickness. In cluster III, the average ring canal size ranged from 1.07±0.14 μm for the M4 ring canals to 2.43 μm for the M1 ring canal. A similar trend was observed in cluster IV (Fig. 4A–C; Fig. S2; 1.21±0.09 μm for M4 ring canals to 2.03 μm for the M1 ring canal). For cluster V, we did not have full coverage of the entire egg chamber volume, so we could not definitively determine the lineage of each ring canal (Fig. S2); however, for those that we could reliably categorize, we found that the M4 ring canals ranged from 1.31 to 1.40 μm, and the M1 ring canal was 3.26 μm in diameter (Fig. 4A–C; Fig. S2). The thickness of the inner rim also varied based on lineage. In region 2b (clusters III and IV), the M1 ring canal contained a thin layer of actin just interior to the electron-dense outer rim; at this stage, the other ring canals (M2–M4) did not show any apparent actin accumulation. By stage 1, an inner rim of actin was obvious in all ring canals, but the thickness of this ring varied significantly based on lineage (Fig. 4A; Fig. S2).

Despite the obvious differences in the absolute thickness of the inner rim, when we compared thickness to ring canal diameter, the differences were not as dramatic, at least for the M1–M3 ring canals. For example, in the stage 4 egg chamber, the thickness of the inner rim was 24–29% of the ring canal diameter for the M1, M2 and M3 ring canals, but it was only 19% of the diameter for the M4 ring canal. This pattern was also observed in the stage 1 egg chamber, where the thickness of the inner rim was ∼21–23% of the diameter of the rings in the M1, M2 and M3 ring canals, but the inner rim was thinner and more heterogenous in the M4 ring canal (ranging from 4 to 12% depending on where the measurement was made). This suggests that the differences in ring canal size and structure observed in the germarium (Fig. 3C; Fig. S2) (Ong and Tan, 2010) are maintained at least through stage 4 of oogenesis. Despite the differences in the size and thickness of the inner rim, we did not observe detectable differences in the thickness of the electron-dense outer region of the ring. The resolution of the SEM data (5–10 nm) did not allow us to measure the nuanced differences in the thickness of the outer region, so additional analysis of this part of the structure will require a more sensitive approach, such as TEM.

### Extensive interdigitations surround ring canals through most of oogenesis

While looking at single sections through the center of the ring canals in the young germline clusters of regions 2a and 3 (stage 1), we were surprised to see evidence of membrane invaginations or interdigitations. Outside the outer rim, we observed additional electron-dense regions along the cell membrane, reflecting areas of stronger cell–cell adhesion (Fig. S3), as has been reported (Fichelson et al., 2010). Near these electron-dense regions, we often observed membrane invaginations, which became more obvious in the stage 1 cluster (Fig. S3, black arrows point to regions of stronger cell–cell adhesion and membrane invaginations). These regions of the membrane were reminiscent of the extensive invaginations, or microvilli, which have been observed by single-section TEM of later-stage egg chambers (Loyer et al., 2015; Tilney et al., 1996). To our knowledge, this is the first time that these membrane structures have been observed in younger germline cell clusters.

**Fig. 5.**
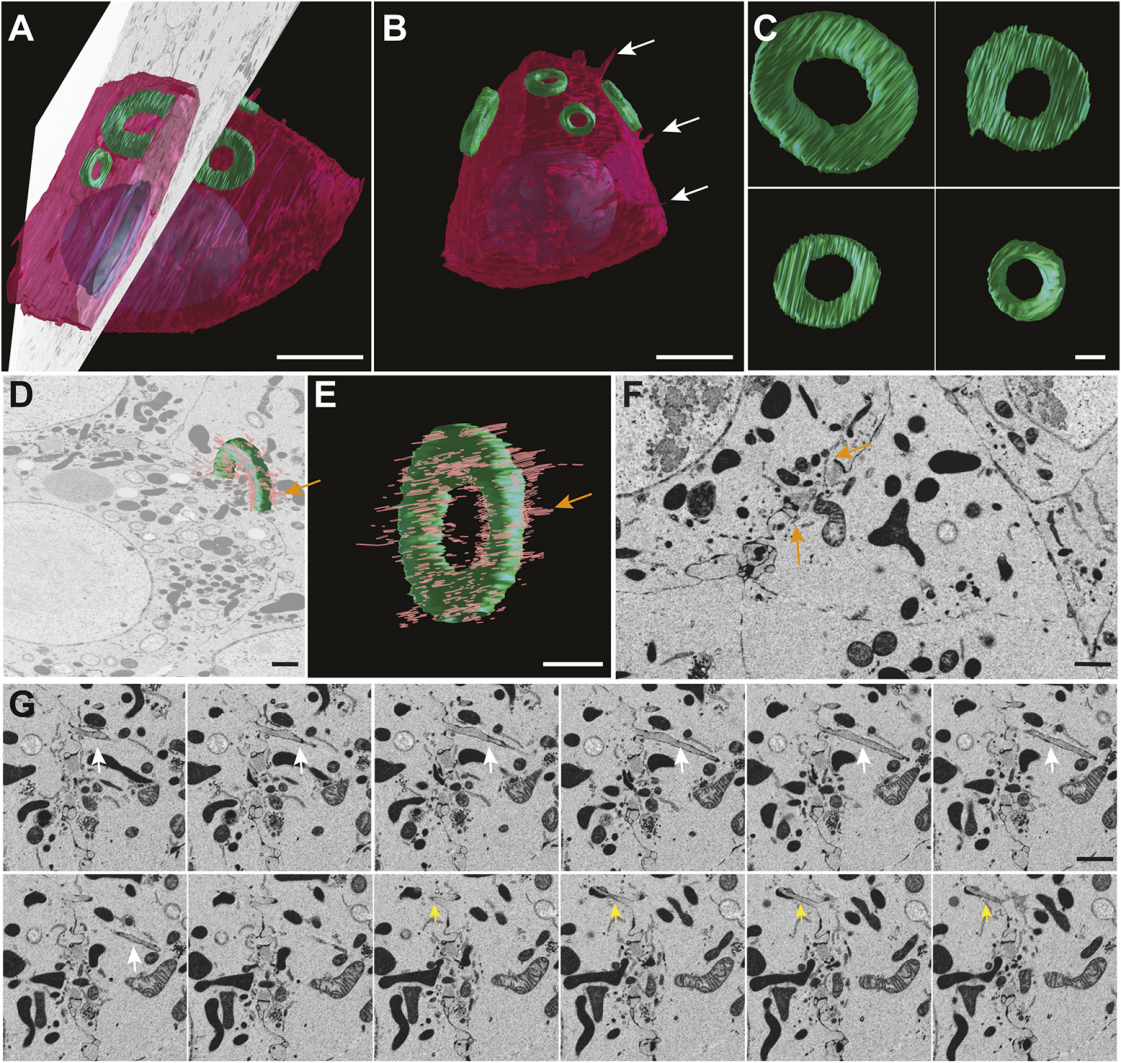
**Interdigitations and ER-like structures.** (A–C) Stage 4 FIB-SEM dataset depicting all membrane interdigitations and ring canals connecting the oocyte and the four posterior nurse cells. Many interdigitations are oriented perpendicular to the nurse cell surface (B, white arrows). (D–F) ER-resembling structures closely align with the inner and lateral surfaces of the ring canal, extending toward the plasma membrane (orange arrows). (G) Multiple sections from the stage 4 egg chamber show that the interdigitations can extend over 4 μm through multiple *z*-sections. Yellow and white arrows highlight two different interdigitations that extend in different directions from the same cell–cell contact. Scale bars: 5 μm (A,B); 1 μm (C–G).

Although the interdigitations were present at these early stages, they became even more elaborate and extensive as the egg chambers developed. To gain further insight into the distribution and orientation of these extensive cell–cell contacts, we performed careful rendering of these structures using the stage 4 FIB-SEM dataset. We created a model that captures all interdigitations present at the membrane of the oocyte and four nurse cells directly connected to it (Fig. 5A–C; Movie 5a at doi:10.6084/m9.figshare.c.8450008). This rendering revealed that interdigitations are present surrounding all four posterior ring canals. We observed many interdigitations oriented perpendicular to the nurse cell surface (Fig. 5B, white arrows); most extended into the nurse cell, but some extended in the opposite direction, into the oocyte. The interdigitations varied in shape; some were long and thin, extending over 4 μm through multiple *z*-sections (Fig. 5G), but others were shorter and broader. There was not an obvious correlation between ring canal lineage and number or abundance of interdigitations.

While performing this analysis, we also observed membrane structures lining many ring canals (Fig. 5D–G; Movie 5b at doi:10.6084/m9.figshare.c.8450008). These membrane structures were initially visible during stages that contained the fusome (Fig. S3A, white arrow), but they persisted even after its breakdown (Fig. S3B, stage 1, white arrows). Although we cannot be certain of their origin or identity, these structures strongly resembled the endoplasmic reticulum (ER), which can be seen in other regions of these images (Fig. 5F,G). Because they were visible in the stage 4 egg chamber, we took the opportunity to learn more about their spatial organization. We found that these structures closely associate with the inner and side portions of the ring canal, flanking it all the way towards the membrane (Fig. 5D–G). Although EM only provides a single snapshot of the tissue, these membrane structures did not appear to be passing through the ring canals; instead, they appeared to cap the inner rim of actin (Fig. 5D–G; Figs S2 and S3, Movie 5b at doi:10.6084/m9.figshare.c.8450008).

### AT-SEM provides a straightforward approach to locating and imaging ring canals and membrane interdigitations within older egg chambers

**Fig. 6.**
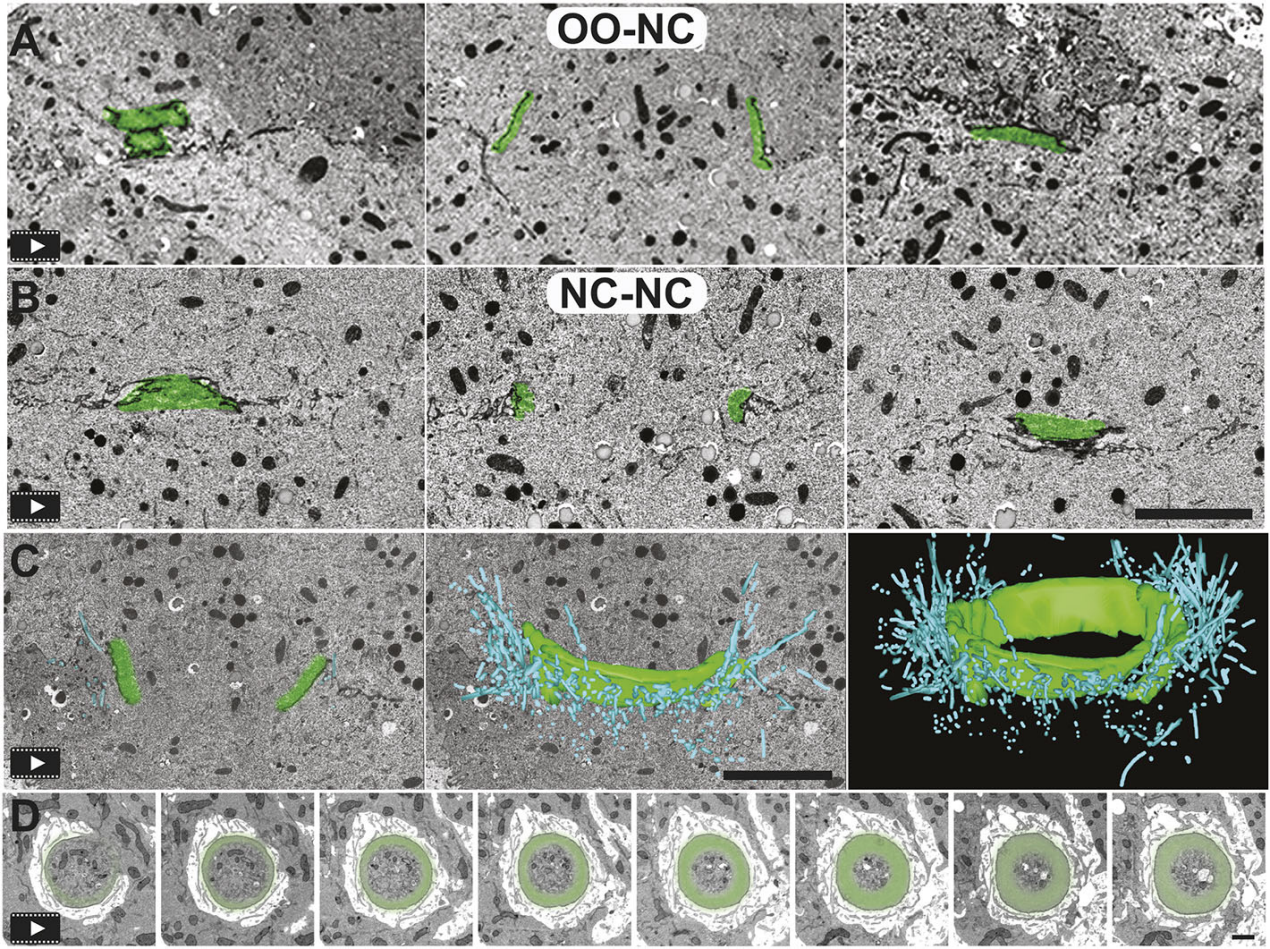
**AT and lateral screening can be used to locate and image ring canals within much larger, later-stage egg chambers.** (A,B) Three sections of the same ring canal connect either a nurse cell to the oocyte (A, OO-NC) or two nurse cells to each other (B, NC-NC). Scale bar: 5 μm. (C) Rendering of the interdigitations (blue) and the actin-rich inner rim (green). Scale bar: 5 μm. (D) A short AT-SEM *z*-series of an imperfectly preserved sample was obtained (5 nm lateral resolution). Membrane tubes, likely pieces of membrane interdigitations, can be seen surrounding the outer region of the ring canal. The dehydration did not dramatically alter the structure of the inner rim (green). Scale bar: 1 μm.

FIB-SEM provided insight into ultrastructural differences in the ring canals (∼1–4 μm in diameter) within developing germline cell clusters and young egg chambers, which occupy a relatively small sample volume (∼50 μm^3^). Ultrastructural analysis of later developmental stages, in which the ring canals are distributed within a much larger volume, cannot be efficiently performed using this approach. AT-SEM provides a more straightforward approach to easily locate the non-uniformly oriented ring canals within a much larger sample volume while maintaining the ability to collect vEM data (Fig. S1A,B). Using this approach, we collected longitudinal sections through an ovariole onto five wafers; each wafer contained 200–300 100 nm-thick sections. Following longitudinal screening of the wafers, we located sections containing ring canals and acquired either higher-magnification tiled images or a sequence of sections for 3D analysis (Fig. 6A,B; Supplemental Material 6 and Movies 6a,b at doi:10.6084/m9.figshare.c.8450008). We then rendered a targeted volume to further visualize the structure of the ring canals and interdigitations in a stage 8 egg chamber. The interdigitations were more abundant at this stage but, as we observed in the stage 4 germline cell cluster, they varied in length and did not show an obvious coordination to their orientation (Fig. 6C; Movie 6c at doi:10.6084/m9.figshare.c.8450008).

Additional insight into the organization of the interdigitations was obtained from another AT-SEM sample in which the tissue structure was disrupted during the preparation process. In this sample, large gaps formed between nurse cells and between the nurse cells and the somatic follicle cell layer; these large gaps provided us with the opportunity to better visualize the interdigitations, which are typically tightly packed around the ring canals. In this imperfect sample, we could clearly see membrane tubes within the tissue gaps. Despite the gaps in the tissue, the actin-rich inner rim and electron-dense outer region structure was still maintained (Fig. 6D; Movie 6d at doi:10.6084/m9.figshare.c.8450008), which is reminiscent of the ring canal detachment phenotype that has been reported in egg chambers with mutations in genes involved in membrane trafficking or adhesion (Coutelis and Ephrussi, 2007; Loyer et al., 2015; Murthy et al., 2005; Murthy and Schwarz, 2004; Oda et al., 1997; Peifer et al., 1993; Starble and Pokrywka, 2018; Tan et al., 2014; Vaccari et al., 2009; White et al., 1998). Additional work is required to determine the composition of this stable structure, especially that of the electron-dense outer region, but it again highlights the value of using vEM to study the ring canal.

## DISCUSSION

The importance of vEM in cellular and developmental biology studies was highlighted recently (Collinson et al., 2023). Here, we have demonstrated that by using two complementary approaches, FIB-SEM and AT-SEM, we gained valuable insights into the 3D ultrastructural changes that the germline ring canals undergo during oogenesis. Our vEM data allowed us to monitor ring canal size, the thickness of the actin-rich inner rim, the structure of the nurse cell membranes, and the early appearance and orientation of extensive membrane interdigitations surrounding the ring canals.

The extensive expansion of the germline ring canals has been one of the reasons that this system has gained popularity in the study of intercellular bridges (Haglund et al., 2011; Robinson et al., 1994), but analysis of ultrastructural differences in ring canal size and thickness was lacking. Our data revealed significant differences in ring canal size and thickness based on developmental stage and lineage. Fluorescence-based analysis of ring canals within the germarium suggested that the degree of contractile ring closure varies based on lineage, with the M1 contractile ring constricting to a diameter of 1.46 μm, whereas the M4 contractile ring constricts to a diameter of 0.79 μm. Furthermore, there is evidence that ring canal growth might differ within the cluster; after the final mitotic division, the M1 canal is the first to initiate the growth phase (Koch and King, 1969; Ong and Tan, 2010). We have recently used fluorescence-based imaging to show lineage-based differences in ring canal scaling; the larger M1 ring canals increase in size more slowly than the smaller ring canals derived from subsequent mitotic divisions (Shaikh et al., 2024). These vEM data demonstrate that the thickness of the actin-rich inner rim differs between ring canals within a cluster (Fig. 4A; Fig. S2), which provides us with a potential mechanism to explain differences in the initiation and progression of ring canal expansion, which could be further explored.

**Fig. 7.**
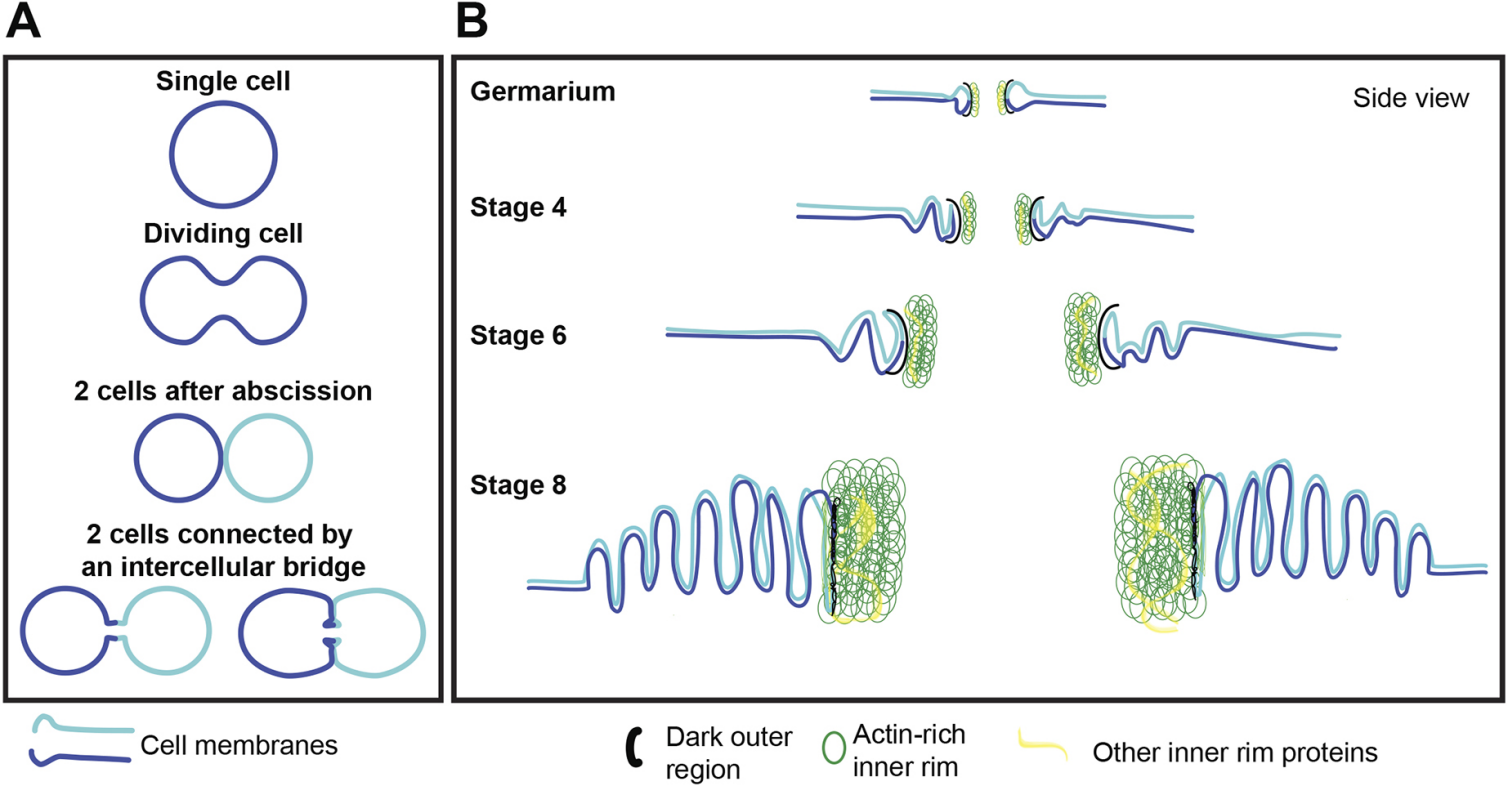
**Model illustrating ring canal formation and expansion.** (A) Following germline cell division, the midbody is remodeled to form an intercellular bridge. (B) As the ring canal expands, the thickness of the inner rim and number of membrane interdigitations increases. Membranes of the neighboring connected cells are represented in dark blue and turquoise.

Our data also provided insights into the structure of the oocyte and nurse cell membranes. Instead of completely separating the daughter cells following division, the midbody is remodeled to generate a stable intercellular bridge (Fig. 7A). As the egg chamber grows, the ring canals expand in diameter (Fig. 7B). Previous TEM-based studies have described ‘microvilli’ that surrounded the ring canals in mid- to late-stage egg chambers (Loyer et al., 2015; Tilney et al., 1996); these microvilli, which are likely stabilized by cadherin-based adhesions, were proposed to anchor the ring canals within the nurse cell membranes (Loyer et al., 2015). Our data revealed that these extensive membrane interdigitations formed earlier than previously reported, at least by region 2b in the germarium (Fig. S3). Although they could be seen along many parts of the nurse cell surface, they were more abundant near the ring canal. At later stages, these interdigitations became even more extensive and pronounced, and our volumetric AT-SEM data demonstrated that their length and orientation was not uniform (Figs 5 and 6; Movies 5a,6a–c at doi:10.6084/m9.figshare.c.8450008). The way that they extended from the ring canal into the oocyte-proximal nurse cell was reminiscent of the actin-based structures that were recently described (Lu et al., 2021). However, these structures could be more similar to the interlocking fingers or lamellipodia-like membrane protrusions that form between epithelial cells (Li et al., 2021; Miyazaki et al., 2023; Senju et al., 2023). These interdigitations are dynamic, and their assembly requires actin regulators, such as the Arp2/3 complex (Li et al., 2021; Senju et al., 2023), which is also required for ring canal expansion and stability (Hudson and Cooley, 2002; Thestrup et al., 2020). Additional high-resolution imaging approaches could be applied to the study of these structures and the associated junctions, as recently described (Janssen and Huveneers, 2024), which might provide mechanistic insight into their role in ring canal expansion or stability. For example, the extensive surface area could increase the number of adhesive junctions that form between nurse cells, which might provide more mechanical strength during tissue growth; alternatively, their formation and retraction could provide a way for the ring canals to easily expand and contract to accommodate the transfer of different volumes of material or react to environmental changes. A recent study demonstrated that overexpression of the inner rim protein HtsRC significantly increased ring canal size (Gerdes et al., 2020); therefore, future studies could determine whether HtsRC overexpression leads to more stable or more extensive interdigitations, which could provide a mechanism to increase ring canal expansion or stabilize these larger ring canals.

In addition to learning more about these intercellular bridges, our high-content datasets can provide ultrastructural insight into many other structures of interest, such as the fusome. Most analyses of the fusome have been performed using a combination of fluorescence microscopy and two-dimensional TEM (de Cuevas and Spradling, 1998; Deng and Lin, 1997; Grieder et al., 2000; Lighthouse et al., 2008; Lin et al., 1994; Lin and Spradling, 1995; Spradling et al., 1997), but our vEM dataset enabled us to generate a 3D model of this structure that provides a level of detail previously unavailable. Our rendering of the stage 2b clusters in the germarium supports a model for the gradual breakdown of the fusome (Fig. 3D), in which the connected membrane network might break down first in the cluster periphery and then later in the central region. This fragmented pattern is consistent with prior live-imaging studies showing that, by region 2b, the fusome was no longer continuous within the cluster (Snapp et al., 2004). Additional vEM analysis of the fusome could further characterize this important germline structure.

### AT-SEM can be used to capture target volumes or to locate hard-to-find structures or cell types

AT-SEM can overcome the specimen size limitations that are encountered with FIB-SEM while providing a workflow to easily identify structures of interest within a large sample volume. In many cases, high-resolution imaging of an entire sample volume is unnecessary, and it would generate a large amount of data that must be stored and managed. With AT-SEM, a lower-resolution map of the sections is generated; from this map, a structure of interest can be quickly identified, and high-resolution imaging of a smaller, targeted volume can be performed. For example, our AT-SEM dataset contained sections of an entire ovariole from the stem cell niche and the mitotic region at the anterior to a stage 10 egg chamber at the posterior. Because our FIB-SEM dataset did not capture the germline stem cell niche, we could easily image and render a small volume of this anterior region of the germarium to fill in this ‘gap’ in our data. Additional sections could easily be imaged to generate a 3D volume of the entire stem cell niche and the mitotic region of the germarium.

A known challenge in EM analysis is finding small, transient structures in a relatively large volume. Our AT-SEM dataset contained an egg chamber at stage 9 of oogenesis, which is the stage at which the border cells are migrating. The border cells are a small group of somatic cells that delaminate from the follicular epithelium at the anterior end of the egg chamber and collectively migrate through the germline cell cluster to the oocyte; these cells will ultimately give rise to the micropyle, which is the site of sperm entry (Rørth, 2002). The border cells have been used as a model for cancer cell metastasis (Stuelten et al., 2018), so learning more about the structure of these cells would improve our understanding of other types of invasive collective migration. Although fluorescence microscopy of both fixed and live samples has revealed much about the dynamics of migration, because of their location deep within a large stage 9 egg chamber, very little ultrastructural information exists.

### The potential of vEM data

vEM data can be generated using different experimental approaches, but each comes with its own set of strengths and limitations. By using a combination of FIB-SEM and AT-SEM, we have been able to capitalize on the strengths and overcome the limitations of each approach. FIB-SEM generates an easily aligned, high-resolution image stack, but because there is an upper limit on the size of the sample that can be imaged, this approach could not be used to monitor ultrastructural changes in larger samples. With AT-SEM, images of much larger sample surfaces can be acquired hierarchically: lower-resolution images can be collected initially to provide a map for screening, and then higher-resolution images can be collected of the relevant structures. Once a region of interest (ROI) is identified in one section, it is straightforward to image sections above and below to capture a small 3D volume. With larger samples and higher-resolution imaging, it is difficult to accurately estimate the dimensions of the acquired data; the germarium is larger than the volume attainable by FIB-SEM, but it constitutes only a small fraction of the stage 9 egg chamber. Therefore, attempting to collect the entire volume of such a large structure at high resolution is impractical. However, because the sections containing the relevant structures of interest can be easily located, volume data that cover only the relevant sections can be acquired; this significantly reduces acquisition time and eases the burden on computational resources. If additional structures become of interest later, the low-resolution map can be used to easily locate and acquire images of a new ROI.

By using EM, the researcher can monitor the location and abundance of the entire morphome of the cell or tissue of interest. Recently referred to as the ‘quiet revolution’ in EM (Collinson et al., 2023), vEM offers ultrastructural insights unattainable through traditional single-section EM techniques. As we have demonstrated, this approach provides not only valuable insight into the structure of interest, but also supplies ultrastructural information about features that were either not expected (such as the membrane lining the ring canal lumen) or not the primary focus (the fusome). Such high-content datasets can foster new collaborations among researchers studying diverse questions within the same system and promote scientific equity by providing access to datasets that might be out of reach for many researchers to generate themselves due to limited funding or access to the necessary equipment or expertise.

## MATERIALS AND METHODS

### Fly stocks, maintenance and husbandry

Flies were maintained on a cornmeal molasses diet at 25°C. The following lines were used: *w^1118^* [Bloomington *Drosophila* Stock Center (BDSC), #3605], *matαTub-GAL4* (BDSC, #7062), *UAS*-*mCherry-RNAi* (BDSC, #35785) and maternal triple driver MTD-GAL4 (*otu-GAL4; nos-GAL4; nos-GAL4*; BDSC, #31777). The genotype of the flies used for the FIB-SEM dataset was *esg-GAL4, UAS-hisCFP, Su(H)nlsGFP; ubi-his-RFP/TM6c*, and the genotype of the flies used for the AT-SEM genotype was *w^1118^, otu-GAL4; nos-GAL4; nos-GAL4*.

### Immunofluorescence and fluorescence imaging

Prior to dissection, offspring from *UAS-mCherry-RNAi* (BDSC, #35785) crossed to *matαTub-GAL4* (BDSC, #7062) (Fig. 2A) were incubated with ground yeast in the presence of males for 48 h at 29°C. Ovaries were fixed in 4% formaldehyde (in PBS) for 15 min, washed with PBS containing 0.1–0.3% Triton X-100, stained with an anti-Hts antibody (1:20, Developmental Studies Hybridoma Bank, 1B1-s). The tissues were then stained with an anti-mouse antibody (1:200, Jackson ImmunoResearch, Alexa Fluor 488 AffiniPure Donkey Anti-Mouse IgG (H+L) cat. no. 715-545-150), Alexa Fluor Plus 647 Phalloidin (1:500, Invitrogen, A30107) and DAPI (1:500, Thermo Fisher Scientific, D3571). The tissues were mounted in Slowfade Diamond Antifade (Invitrogen, S36972) and imaged on a Nikon Ti2-E Inverted microscope equipped with a Yokogawa CSU-X1 spinning disk and Hamamatsu ORCA fusion camera with a 100× Plan Apo VC objective (NA 1.4).

### EM sample preparation

The egg chambers [FIB-SEM genotype: *esg-GAL4, UAS-hisCFP, Su(H)nlsGFP; ubi-his-RFP/TM6c*; AT-SEM genotype: *w^1118^, otu-GAL4; nos-GAL4; nos-GAL4*] were prepared and fixed using a previously described procedure (Kolotuev, 2014; Loyer et al., 2015). Briefly, the egg chambers were dissected in PBS and immediately fixed in 2% paraformaldehyde and 2.5% glutaraldehyde in 0.1 M phosphate buffer for 2 h at ambient temperature. Samples were postfixed with 1% osmium tetroxide [Electron Microscopy Sciences (EMS), 19152] in 1.5% potassium ferrocyanide (EMS, 26604). Samples were then incubated in 1% aqueous uranyl acetate solution and dehydrated with increasing concentrations of ethanol (30%, 50%, 70%, 90%, 3×100%) and infiltrated with epon-araldite mix (EMS, 1420) resin through increasing percentages of resin with ethanol (Burel et al., 2018; Kolotuev, 2014).

### Block preparation and ultramicrotomy

Samples were flat embedded in epon-araldite and polymerized at 60°C for 48 h, following the procedure described previously (Kolotuev, 2014). The flat embedding is crucial for the later orientation of the blocks for sectioning (Fig. 1C; Fig. S1A). After polymerization, samples were trimmed to remove empty resin and to expose the area of interest with a 90° diamond trim tool (Diatome, Switzerland) and mounted on a Leica UC6 microtome for trimming.

#### FIB-SEM sample preparation

Samples were trimmed and attached to aluminum stubs using a silver epoxy mix (Kizilyaprak et al., 2019) while carefully orienting the surface of interest facing the edge. The samples were oriented perpendicular to the sectioning plane to ensure that the smallest dimension was created for imaging (∼60 μm wide, Fig. S1).

#### AT-SEM sample preparation

Samples for AT-SEM were sectioned using the ultramicrotome as described (Burel et al., 2018; Franke and Kolotuev, 2021; Kolotuev, 2014). Briefly, polymerized flat blocks were secured inside the ultramicrotome holder and carefully oriented perpendicular to the sectioning plane with the overall ROI protruding slightly from the tip (Fig. S1). After trimming, a dedicated ats knife (Diatome) was used to generate the sequences of consecutive sections, or arrays. Typically, 50–200 sections were collected per ribbon with 50–150 nm thickness. Arrays were transferred to 2×4 cm bits of a silicon wafer (Ted Pella, 16015), and wafers were air-dried and placed in an oven at 60°C for 1 h for additional adhesion.

### EM acquisitions

For both types of acquisitions, the HELIOS Nanolab scanning microscope (Thermo Fisher Scientific) was used. Images were acquired with a backscattered electron detector (mirror detector) at an accelerating voltage of 2 kV and probe current of 0.8 nA at a distance of 2.5 mm from the detector. To achieve a TEM-like appearance, the micrographs were collected in the inverted mode.

#### FIB-SEM

The acquisitions were performed automatically and piloted by the Slice and View program (Thermo Fisher Scientific), as previously described (Kizilyaprak et al., 2019). The 10.2 nm pixel resolution stack (germarium) was acquired at 25 nm cutting thickness (raw data pixel size of 0.0102×0.0102×0.025 μm^3^), and the 9.9 nm pixel resolution stack (stage 4 egg chamber) was acquired at 20 nm cutting thickness (raw data pixel size of 0.0099×0.0099×0.020 μm^3^).

#### AT-SEM

The acquisitions were performed using the Maps program (Thermo Fisher Scientific) in semi-automatic acquisition mode as single frames or stitched mosaic panels to cover more extensive regions like those previously described (Franke and Kolotuev, 2021). After lateral screening for specific features of interest, mid-range-resolution (∼20 nm pixel size) images were acquired to identify the ROI (Burel et al., 2018) more precisely. Then, the ROIs were collected with higher resolution (5 nm pixel size) as single images or tiles of consecutive images. Acquisition parameters were adjusted based on the dataset, varying between low-resolution and high acquisition speed parameters during screening, and high-resolution and low acquisition speed during detailed maps and stack acquisition.

### Alignment, segmentation and image analysis

To align and segment the datasets, a combination of different programs was used, including Fiji TrakEM2 (Cardona et al., 2012), IMOD (Kremer et al., 1996), Amira (Thermo Fisher Scientific) and Adobe Photoshop. The specific approach was selected based on the quality of the stack and the required alignment precision. FIB-SEM datasets do not require much alignment as the image stacks are obtained with small *z*-steps and little distortion. With AT-SEM, there is some distortion due to the sectioning limitation, thicker *z*-sections and overall larger acquisition area/volume. In this case, 3D alignment is more challenging and, in some cases, multiple programs were required to obtain a well-aligned stack.

Manual segmentation of the samples was done using the IMOD 3dmod program; the contours were traced by hand and extrapolated to generate the 3D model. The recognition of the structures of interest was done by the operator. The structures that could not be traced manually due to their complexity were rendered using the isosurface function of 3D mod (https://bio3d.colorado.edu/imod/doc/3dmodHelp/modvIsosurface.html). This tool uses a selected sub-volume to generate surfaces in the model from regions where the intensity value crosses a threshold.

## Supplementary Material



10.1242/joces.264713_sup1 Supplementary information
