# Supplementary material for: Complementary volume electron microscopy-based approaches reveal ultrastructural changes in germline intercellular bridges
Source: J Cell Sci. 2026 May 26;139(10):jcs264713. doi: 10.1242/jcs.264713 (PMC13286353; doi:10.1242/jcs.264713)
Supplement: Supplementary information [file joces-139-264713-s1.pdf]

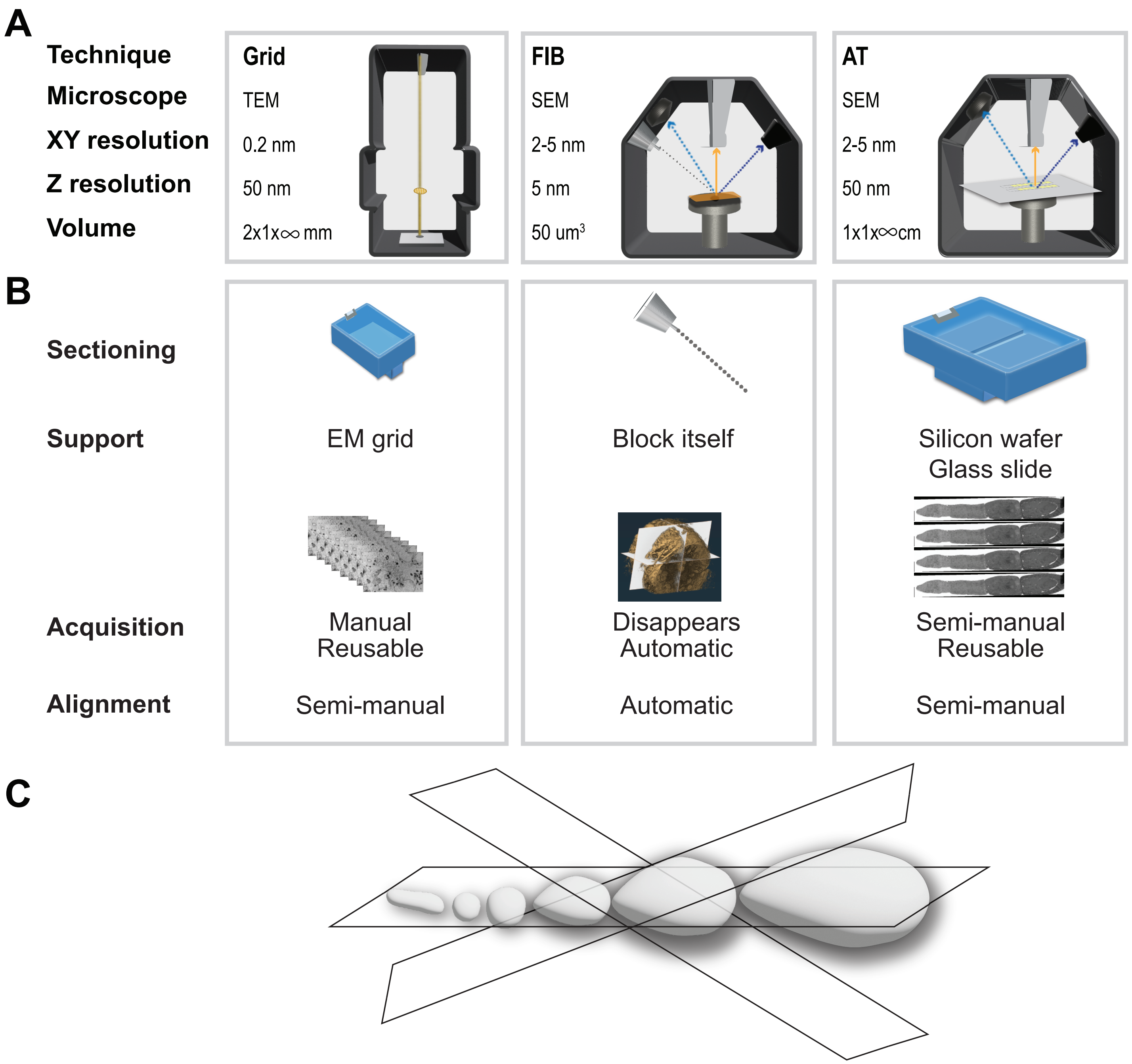

Journal of Cell Science • Supplementary information

Fig. S1. Sample preparation, acquisition, alignment, and analysis for different types of vEM. All described techniques are performed at room temperature with adaptations in preparation techniques based on the type of the sample. (A) Illustration of TEM, FIB-SEM, and AT-SEM approaches. Within each box is an indication of the XY and Z resolution and the potential volume that can be captured by each vEM approach. The TEM microscope uses an electron beam that penetrates through the sample, projecting the image on the camera below. FIB and AT use SEM technology, where only the surface of the sample is scanned by the electron beam. Detectors collect scattered electrons that are “reflected” from the surface after exposure to an electron beam. The difference between FIB and AT is how the external layer of the sample is exposed to the surface. In FIB, the surface is exposed by the gallium ion beam operated inside the SEM sample chamber and subsequently imaged by the scanning electron beam. For AT, embedded samples are sectioned to produce ribbons of sequential sections (arrays) that are transferred to a solid support. Lateral scanning can then be used to identify sections of interest for SEM-based imaging. (B) For TEM, the samples are sectioned on an ultramicrotome with a diamond knife and transferred to support grids (round, 3mm diameter, thin metal foil). The maximum area of the sample is limited by these dimensions. The thickness of the sections varies between 50 and 300 nm and is defined by the penetration capacity of the electron beam. The resulting images provide a very high resolution (~ 0.2 nm), but higher magnification images result in a smaller field of view. For FIB-SEM, minimal manipulation is required before introducing the sample into the SEM sample chamber. The block surface is milled directly inside the SEM chamber and the images are collected from the newly exposed surface. The destructive nature of the acquisition prevents modification or reacquisition of the images once acquired. FIB removes 5-50 nm of the surface before capturing a SEM image, with the cycle continuing to create a nearly perfect 3D stack through a relatively small sample volume. The stack of images is easy to align, and isotropic resolution can be achieved. For AT-SEM, blocks are sectioned using a diamond knife, creating a sequence of sections (arrays), which is transferred to a large support, such as a wafer or a coated glass coverslip. The physical sectioning by the diamond knife permits the processing of a relatively large area (> 1 mm^2) and a nearly unlimited z depth. The primary strength of the technique is the ability to use lateral screening of the arrays to efficiently locate

Journal of Cell Science • Supplementary information

the ROI, which can then be selectively imaged at higher resolution. With tiled acquisition there is no real upper limit to the area that can be acquired; further, sections are stable and can be repeatedly imaged at a range of resolutions. z resolution is limited by physical sectioning to ~50 nm. The alignment and segmentation steps of the workflow are frequently performed with the same program. The alignment can be performed automatically with the help of the program's algorithms or manually by adjusting the consecutive images individually. The segmentation depends on the scope of the desired model and the abundance and complexity of the structures; it can be done manually or with automation. Serial sections are well-organized on wafers 1,2,3, and 5, but was disrupted on wafer 4; the order of the sections can be re-established during imaging or alignment. Scale bar is 1 mm. (C) Different sectioning orientations result in the highly variable appearance of the same structure, highlighting the importance of using vEM to study these structures.

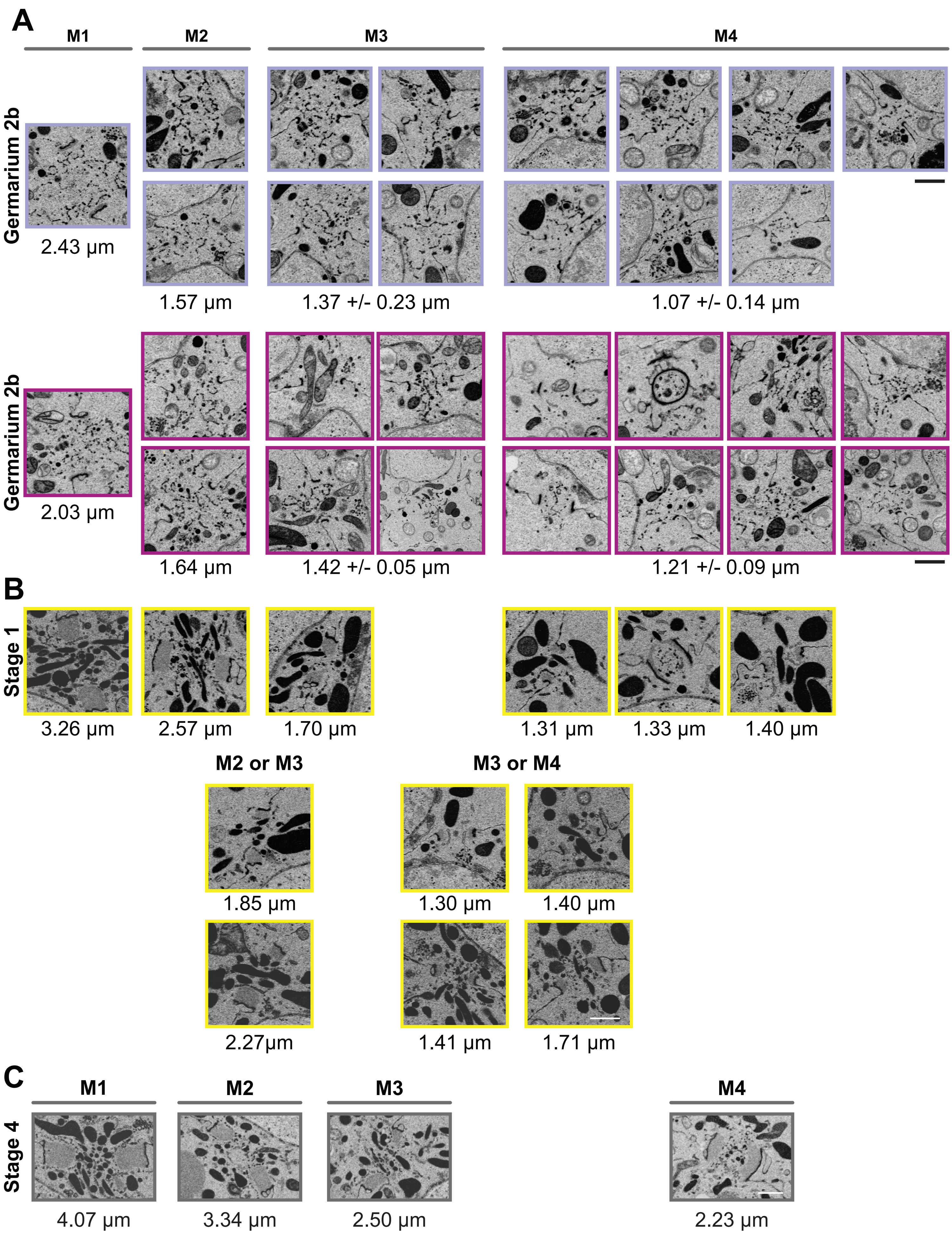
A
M1
M2
M3
M4
Germarium 2b
2.43 µm
1.57 µm
1.37 +/- 0.23 µm
1.07 +/- 0.14 µm
Germarium 2b
2.03 µm
1.64 µm
1.42 +/- 0.05 µm
1.21 +/- 0.09 µm
B
Stage 1
3.26 µm
2.57 µm
1.70 µm
1.31 µm
1.33 µm
1.40 µm
M2 or M3
M3 or M4
1.85 µm
1.30 µm
1.40 µm
2.27µm
1.41 µm
1.71 µm
C
M1
M2
M3
M4
Stage 4
4.07 µm
3.34 µm
2.50 µm
2.23 µm

Fig. S2. Single-plane FIB-SEM images show the differences in ring canal size, thickness, and orientation within each cluster. Images were cropped to highlight the germline ring canals within each cluster. (A) The two clusters from region 2b (purple and pink) were sufficiently covered to determine mitotic division of origin for all ring canals. (B) For the stage 1 cluster (yellow), division of origin could be definitively assigned for about half of the ring canals, and for the other half, origin could be narrowed down to one of two types. (C) The older, stage 4 egg chamber (grey) was not completely captured, so only the four posterior ring canals directly connecting to the oocyte could be definitively characterized. Scale bars are 1 µm.

Journal of Cell Science • Supplementary information

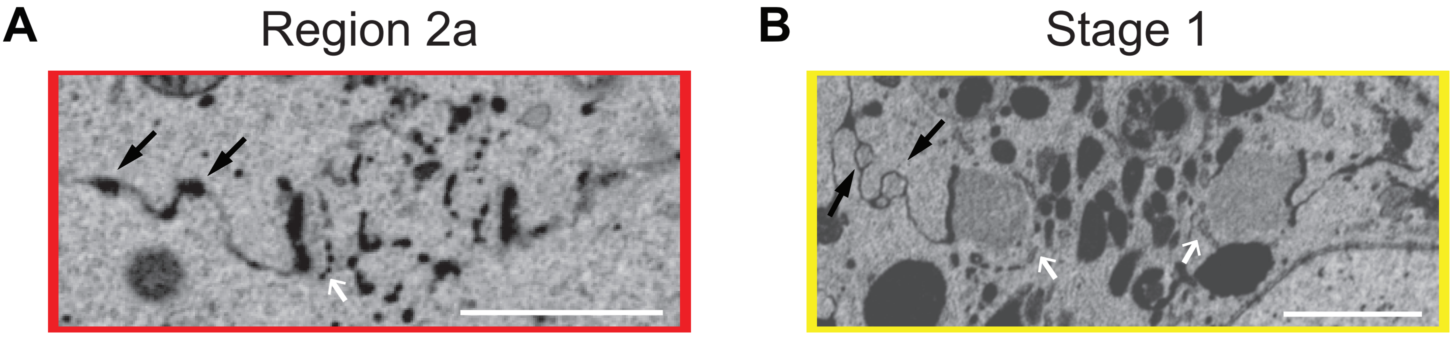

Fig. S3. Interdigitations appear earlier than previously reported. Single-section images taken through the center of ring canals in early germline clusters from region 2a (A) and region 3/stage 1 (B) exhibited membrane invaginations or interdigitations. Additional electron-dense areas were evident along the cell membrane, indicating regions of enhanced cell–cell adhesion near these interdigitations (A,B black arrows). ER-resembling structures were seen lining the ring canals (A,B white arrows). Scale bars are 1µm. We did not have enough coverage of the region 2a cluster (red) to determine the lineage of this ring canal; the example from the stage 1 cluster (B) is an M1 ring canal..

Journal of Cell Science • Supplementary information

Movie 1a. Movie of entire FIB-SEM image stack through the germline clusters within the germarium, which are rendered in Fig. 3.

Available at https://doi.org/10.6084/m9.figshare.c.8450008

Movie 1b. Movie of entire FIB-SEM image stack through part of the stage 4 egg chamber.

Available at https://doi.org/10.6084/m9.figshare.c.8450008

Movie 2. Movie of a FIB-SEM serial section alignment through the ring canal presented in Fig. 2E.

Available at https://doi.org/10.6084/m9.figshare.c.8450008

Movie 3a. A series of aligned sequential EM sections was obtained using FIB-SEM, focusing on the germline region. Different clusters were manually segmented with separate colors for each cluster. Ring canals for each cluster were rendered using the isosurface feature of IMOD.

Available at https://doi.org/10.6084/m9.figshare.c.8450008

Movie 3b. A series of sequential EM sections were obtained using FIB-SEM, focusing on the stage 2b cluster. The segmentation highlights the complexity of the fusome in the germline (white) and its interaction with the ring canals connecting different germline cells within the cluster.

Available at https://doi.org/10.6084/m9.figshare.c.8450008

Movie 5a. A series of aligned sequential EM sections was obtained from a stage 4 egg chamber using FIB-SEM. Oocyte cell membrane and ring canals were manually segmented.

Available at https://doi.org/10.6084/m9.figshare.c.8450008

Movie 5b. A series of aligned sequential EM sections was obtained from a stage 4 egg chamber using FIB-SEM. Sequence cropped to highlight the ER-resembling structures that are found near one ring canal.

Available at https://doi.org/10.6084/m9.figshare.c.8450008

Journal of Cell Science • Supplementary information

Supplemental Material 6. A composite of tiled images acquired with 5 nm resolution. Uncompressed image represents 1101x266 cm.

Available at https://doi.org/10.6084/m9.figshare.c.8450008

Movie 6a. Aligned serial sequence of the oocyte-nurse cell (OO-NC) ring canal from the AT acquisition shown in Fig. 6A.

Available at https://doi.org/10.6084/m9.figshare.c.8450008

Movie 6b. Aligned serial sequence of the nurse cell-nurse cell (NC-NC) ring canal from the AT acquisition shown in Fig. 6B.

Available at https://doi.org/10.6084/m9.figshare.c.8450008

Movie 6c. Aligned serial sequence of the OO-NC ring canal from the AT acquisition shown in Fig. 6A. The movie combines the EM sequence and the subsequent segmentation of the outline of the ring canal (green) and the portions of the interdigitations (light blue) as shown in Fig. 6C.

Available at https://doi.org/10.6084/m9.figshare.c.8450008

Movie 6d. Serial sections through the ring canal from the imperfectly prepared sample shown in Fig. 6D.

Available at https://doi.org/10.6084/m9.figshare.c.8450008
