# Supplementary material for: Complementary volume electron microscopy-based approaches reveal ultrastructural changes in germline intercellular bridges
Source: J Cell Sci. 2026 May 26;139(10):jcs264713. doi: 10.1242/jcs.264713 (PMC13286353; doi:10.1242/jcs.264713)
Supplement: Supplementary file 1 [file joces-139-264713_review_history.pdf]

Complementary volume electron microscopy-based approaches reveal ultrastructural changes in germline intercellular bridges

Irina Kolotuev, Sophie Khazanov, Abigayle Williams, Caroline Kizilyaprak, Stephanie Pellegrino and Lindsay Lewellyn
DOI: 10.1242/jcs.264713

Editor: Guillaume Jaquemet

Review timeline

Submission to Review Commons:	27 February 2025
Submission to Journal of Cell Science:	9 January 2026
Editorial decision:	6 February 2026
First revision received:	7 April 2026
Accepted:	8 April 2026

Reviewer 1

Evidence, reproducibility and clarity

Summary

The possibility of observing 3D cellular organisation in tissues at nanometre resolution is a hope for many cell biologists. Here, the authors have combined two volume electron microscopy approaches with scanning electron microscopy: Focused Ion Beam (FIB-SEM) and Array Tomography (AT-SEM) to study the evolution of the shape and organisation of cytoplasmic bridges, the 'ring canals' (RCs) in the Drosophila ovarian follicle that connect nurse cells and oocyte. This type of cytoplasmic link, found in insects and humans, is essential for oocyte development. RCs have mainly been studied using light microscopy with various markers that constitute them, but this approach does not fully capture an overall view of their organization. Due to their three-dimensional arrangement within the ovarian follicle, characterizing their organization using transmission electron microscopy (TEM) has been very limited until now.

This v-EM study allows the authors to document the evolution of RC size and thickness during the development of germline cysts, from the germarium to stage 4, and potentially beyond. This study confirmed previous findings, namely that RC size correlates with lineage:

the largest RC is formed after the first division, while the smallest is formed during the last division.
Furthermore, this work allowed a better characterisation of the membrane interdigitation surrounding the RCs.
In addition, the authors highlight the important potential of v-EM for further structural analysis of the fusome, migrating border cells and the stem cell niche.

Major comments

- The output of this work can be divided into two parts.
First, this work presents a technical challenge, involving image acquisition by volume electron microscopy and manual 3D reconstruction of the contours of the membranes, nuclei, RCs, and fusome in different cysts at different stages.
Secondly, this work is based on a structural study of the RCs and their associated membranes. This work is descriptive but important, although the results largely confirm previous findings, both for the structure of the RCs and their relationship to the division sequence of the cyst

© 2026. Published by The Company of Biologists under the terms of the Creative Commons Attribution License (https://creativecommons.org/licenses/by/4.0/).

cells, and for the organisation of the membranes around the RCs.

- Very interestingly, the authors report the spatial characterisation of membrane structures associated with and close to CRs that have already been identified (Loyer et al.). However, their characterisation is somewhat incomplete, as it lacks quantified data - how many CRs were analysed? and, above all, the characteristics of these membranes, their length and orientation according to their position and their connection in the lineage - these data could be obtained from the VEM data already collected and would be an important addition to the RC structural analysis in this work.

In line with this, the authors importantly report the presence of an ER-like membrane structure lining the RCs. First, it would be nice to have statistics to support the observation of how many RCs..? Secondly, does this ER membrane structure vary according to the position of the RC in the cyst, are they related to the RC lineage?

The addition of graphs showing the quantitative data with statistics in the figures would improve understanding of the results. This is particularly the case for the characterisation of RCs according to the stage of cyst development, as shown in Figure 3. This also applies to the characterisation of RCs within a cyst and the relationship between RC size and lineage, as shown in Figure 4, and to the characterisation (thickness) of the inner part of the RC.

-The part on the structural analysis of the fusome is interesting but still secondary to the characterisation of the RCs. This part should be moved to the results and figures after the various parts concerning the RCs.

Minor comments

-The distribution of the fusome in Figure 2 is difficult to see with Hts labelling and does not really correspond to the schematic, especially in regions 2a and 2B.

-In panel C of Figure 2, it is a little disturbing that the legend is directly on the image of RC. It hides some information about the images and could be placed at the bottom of the panel. This also the case for the panel G.

With figure 3B, it would be good to highlight the position of cyst.

Significance

As mentioned above, this work can be divided into two parts.

The part corresponding to the acquisition of images by volume electron microscopy and manual 3D reconstruction is new and a great source of valuable information.
The part related to the spatial characterisation of the RC is important, but corresponds more to an extension and reinforcement of previously available information than to the contribution of significant new insights.

I think it will be of great interest to an audience interested in Drosophila oogenesis.

Reviewer 2

Evidence, reproducibility and clarity

This study presents a high-resolution volumetric analysis of germline ring canals (RCs) during Drosophila oogenesis. By combining two complementary electron microscopy techniques-Focused Ion Beam Scanning Electron Microscopy (FIB-SEM) and Array Tomography Scanning Electron Microscopy (AT-SEM)-the authors compare RC structural features at different developmental stages, ranging from the relatively small germarium to the significantly larger, later-stage egg chambers.

© 2026. Published by The Company of Biologists under the terms of the Creative Commons Attribution License (https://creativecommons.org/licenses/by/4.0/).

At early stages of oogenesis, FIB-SEM analysis confirms that the average RC size increases progressively with cyst development, in agreement with previous studies. The authors further show that lineage reliably predicts RC size (an observation previously reported, but here identified at an earlier stage in region 2a) and, importantly, that the thickness of the actin rim can also be predicted by lineage (reported here for the first time, at stage 1). FIB-SEM analysis also enables a clear delineation of the fusome, allowing for detailed characterization of its assembly and disassembly. Notably, the authors report, for the first time, structural evidence of ER-like membranes capping the inner rim of actin RCs.

At later developmental stages, AT-SEM analysis reveals that the microvilli observed by FIB-SEM evolve into extensive interdigitations extending beyond the outer rim in mid-stage egg chambers, a structural feature detected earlier than previously reported. Moreover, by analyzing a sample in which tissue organization was disrupted during preparation, the authors demonstrate that these interdigitations preferentially occur in proximity to the RC. In addition to RC analysis at later stages, the authors use AT-SEM to readily identify small cell populations, such as the germline stem cell niche and border cells, and provide high-resolution volumetric EM data for these structures.

MAJOR COMMENT

My main comment is that we don't learn much new about the biology of these ring canals. The results primarily confirm findings from previous studies using conventional electron microscopy. One particularly interesting biological question, which is briefly mentioned in the text, is whether the oocyte is the cell that inherits the majority of the fusome. Since the authors are able to reconstruct the fusome using their data, they could measure the fusome volume in each cell (especially in the two pro-oocytes) and investigate whether the cell with the larger fusome ultimately becomes the oocyte. This question has been discussed for some time, and recent studies have proposed opposing models based on fusome volume to explain how the oocyte is selected among the 16 sister cells (Nashchekin et al., Science, 2021; Barr et al., Genetics, 2024).

MINOR COMMENTS

• The fluorescent markers used in the fly stocks are neither described in the Materials and Methods section nor depicted in the figures.

• The authors should quote (Nashchekin et al., Science, 2021) when mentioning unequal partionning of the fusome (p4) and oocyte determination (p12).

• P11-12, when mentioning electron dense regions reflecting strong cell-cell adhesion, the authors could refer to (Fichelson et al. Development, 2010), where AJ have been described around ring canals.

• Figure 2A: The schematic diagram (4th line) is not explained in the figure legend.

• Figure 2D: Please clarify whether the RC stage shown corresponds to stage 1 or stage 10, as indicated in panel 2E. Alternatively, are these examples representing the minimum and maximum RC sizes observed across the entire dataset?.

• Figure 5D: Please specify which panel in 5B this corresponds to.

• Figure 5E: Please specify which panels in 5B this corresponds to. The two green boxes are not defined. Why is there a grey background under the ovariole assembly?

• Figures 5G, 5H: Does panel 5G correspond to the left green box in 5E, and 5H to the right green box in 5E? Please clarify.

• Figure 6: The figure title is not on the same page as the figure itself.

© 2026. Published by The Company of Biologists under the terms of the Creative Commons Attribution License (https://creativecommons.org/licenses/by/4.0/).

• Figure 6A: The black box marking the germarium is not defined.

• Figure 6B-E: The arrows point to long interdigitations. However, arrowheads (which are not mentioned in the legend) appear to indicate the RC outer rim. Please specify this clearly in the figure legend.

Significance

I am not an expert in electron microscopy, so I cannot comment in detail on these techniques, but they appear to bridge the gap between conventional EM and optical microscopy in terms of resolution, user-friendliness, and other aspects. This is technically interesting, although these EM approaches have been previously described and applied. The images and movies are beautiful and clearly presented.

My main comment is that we don't learn much new about the biology of these ring canals. The results primarily confirm findings from previous studies using conventional electron microscopy. One particularly interesting biological question, which is briefly mentioned in the text, is whether the oocyte is the cell that inherits the majority of the fusome. Since the authors are able to reconstruct the fusome using their data, they could measure the fusome volume in each cell (especially in the two pro-oocytes) and investigate whether the cell with the larger fusome ultimately becomes the oocyte. This question has been discussed for some time, and recent studies have proposed opposing models based on fusome volume to explain how the oocyte is selected among the 16 sister cells (Nashchekin et al., Science, 2021; Barr et al., Genetics, 2024).

Reviewer 3

Evidence, reproducibility and clarity

Kolotuev et al. used two volume-based electron microscopy based approaches to identify, segment, and document the changes in intercellular bridges, or ring canals, in early egg chambers of the fruit fly, Drosophila melanogaster. Using array tomography and focused ion beam scanning electron microscopy, Kolotuev et al., provide a high resolution and content rich lineage analysis of ring canal size, shape and orientation among early and late egg chambers. Their analysis included parameters such as the presence and shape of the fusome, the recruitment of actin to the inner ring, and development of membrane fingers that presumably spatially stabilize such structures. Last, Kolotuev and co-authors highlight additional aspects of their dataset including a reconstruction of the border cell cluster in stage 9 egg chambers. The data presented are a treasure trove of the ultrastructural features of the developing dipteran germline and subsequent ovarian follicle development. The data presented represent the highest resolution 3D dataset available and thus are a valuable worthwhile contribution to the field. My overall impression is that this paper sits intellectually between a valuable method and a loose experimental manuscript. This critique is not requesting additional experimental evidence because the data are unique and are the foundation for a new experimental paradigm. But there is not sufficient detail presented to be a full method, nor any hypothesis testing to be considered experimental. I suggest the authors consider amplifying their methods in detail and then note that using these methods provide a foundation for additional future investigations (as mentioned in the discussion).
Problems with data interpretation and presentation should be addressed before publication. Below are the major and minor concerns that I believe need to be considered.

Major comments:

- In general images in figures are thought provoking, however changes to figure layout and design should be considered to better highlight the results. For instance, I don't know how to follow figure 1a. The arrow leads from a whole ovary to an ovulated egg with an ovariole strand connecting the two. What is the purpose of the arrow? Is it to represent time? And why is the mature egg in the figure when no data regarding this stage is presented. The authors should consider removing the mature egg and helping the reader understand that the ovariole

© 2026. Published by The Company of Biologists under the terms of the Creative Commons Attribution License (https://creativecommons.org/licenses/by/4.0/).

is a subset of the whole ovary. They might do this by putting a box around a single ovarile in the whole ovary to indicate their ovariole illustration. Several other figures have similar problems. Throughout the authors used black and white arrows on black and white EM data and these arrows were lost. Color should be considered to effectively point out what they want the reader to see.

- Can the authors provide additional information for the genotypes used? For instance the Cherrio-YFP (which might affect actin). When what this used and can the authors provide information on how this affected the data between when it was used and when it was not used. Additionally, why was analysis done in transgenic flies over fully wild-type?
Figure 1 seeks to lay out the ovary system and narrow the reader into the stages that will be analyzed in subsequent figures. Figure 1B is meant to show the types and kinds of electron microscopy, however lacks a full detailed description and legend for each of the colored arrows. And to that fact, so does figure S1. The authors need to provide additional information so the reader can glean what the authors point they are trying to convey. In addition, the authors might add pros and cons to each. I know this was attempted in S1, but did not fully come across.
Figure 1 and 2 seek to set up both the biological and technical system to be understood. The authors might consider combining the two figures and eliminate elements that don't represent a result of any kind (Figure 1B, 2B, 3D and 3F). Or more fully explain the result and point they are trying to make with these illustrations. I fully understand and appreciate what they are trying to get across, but it does not come across clearly. For example, I don't know how figure 2B effectively gets across the point that rotation of the image has an effect on how it is sliced and segmented in EM data. Not sure it is necessary. Furthermore, what is the bottom panel with a green ring canal supposed to allow us to interpret or conclude? The same for 3D and F. The result in 3E is far more interesting and should be two panels that emphasize the growth characteristics between young and old rings or those of M1 and M4.

- The HTS and actin stain in figure 2A overlap significantly and obscure the fusome staining. Can the authors confirm that there is no bleed through in their staining and imaging procedure?

- The data in Figure 2C are critical to showing the z-resolution enhancement of sectioned EM. However, the use of green psuedocolor only in one panel is confusing. Can the authors duplicate the whole panel and provide one without and one with psuedocolor? This would be ideal for fully orienting the reader to the sectioning and setting them up to understand the rest of the figures.

The results section for figure 2 does outline the results presented. For example, the germarium contains syncytia of differing stages and ring canals with intervening fusomes... It does more to talk about the pros and cons of different technical aspects and their difficulty This should be saved for the rationale or the discussion. Rather the section should outline the results presented.

- I appreciate the color coding of the differentially segment cysts in Figure 3. The color coding helped orient me to which cysts were being evaluated. However I found the lack of detail bothersome. For instance, which ring canals are in the two panels of D? Are they M1 or M4? Also, the presentation of ring canal size and distribution should be presented in a graph. Statistics are not necessary, but a dot-plot would go a long way to presenting the result. Two plots can add value, one in which the ring canals for each phase is shown, and the other is the distribution of sizes for each cyst. Lastly, the results section for figure 3 interprets the membrane bound vesicles in the ring canal as "ER-like". This should be removed since they neither look ER-like to me, nor have been shown to be ER in the data.

- Figure 4A is not called out specifically in the results and thus should be interpreted or removed from the figure.

- Figure 5 was confusing. I understand the authors wanted to show the wafer and the ribbons, however, this is not a result and does not offer any interpretation of a result and is thus confusing on why it is in the figure. If this were a method paper, I would understand its

© 2026. Published by The Company of Biologists under the terms of the Creative Commons Attribution License (https://creativecommons.org/licenses/by/4.0/).

presence.

- Can the authors comment on the shape of the nuclei in older egg chambers? They are not round at all. I am interested in whether this is a fixation artifact or the real ultrastructure of the nuclei. Of the border cell nuclei for instance. If it is an artifact, this should be added to the discussion.

- Although data from "imperfect" samples is interesting, consider relegating Figure 6 to the supplement section, as it takes away from the pre-existing narrative flow established in the paper.
Interpretation of the data throughout the results should be left to the discussion section. For instance, interpretation of Figure 4 results on page 14 beginning with "these data demonstrate the importance...". The importance is not related to the result, but rather discussion of past and future studies. In another example, Figure 5I is introduced and discussed in the results section on page 15, second whole paragraph with an overall introduction/discussion on junctions, which convolutes the actual result. Discussion of future studies or how structures like the novel membrane fingers should be viewed in a larger biological context, should not be in the results.

Minor comments:

- Remove words such as "pseudo-timelapse", they invoke precision on a point that is imprecise.

- Re-consider the acronyms for ring canal and egg chamber.

Significance

The present manuscript is a technical advance in the field. The use of serial EM imaging with two separate modalities, on what is considered to be a challenging problem in the field, represents a useful technical advance. Light microscopy has thus far limited the resolution to which we can understand the spatial organization and the cellular features there in that regulate germline development. This manuscript brings to bear two serial EM methods to begin approaching this problem. The audience for this work are those working at the forefront of understanding germline architecture and development. I make these statements as an expert in live and super resolution of fruit fly egg chamber development, in addition to having performed 3D SEM in past works.

<u>Author response to reviewers' comments</u>

We would like to thank the three reviewers for their careful reading of our manuscript and suggested modifications. We have incorporated their suggestions as described below; these changes have significantly improved the structure and focus of the manuscript.

Reviewer #1 (Evidence, reproducibility and clarity (Required)):

<u>Summary</u>

The possibility of observing 3D cellular organisation in tissues at nanometre resolution is a hope for many cell biologists. Here, the authors have combined two volume electron microscopy approaches with scanning electron microscopy: Focused Ion Beam (FIB-SEM) and Array Tomography (AT-SEM) to study the evolution of the shape and organisation of cytoplasmic bridges, the 'ring canals' (RCs) in the Drosophila ovarian follicle that connect nurse cells and oocyte. This type of cytoplasmic link, found in insects and humans, is essential for oocyte development.

RCs have mainly been studied using light microscopy with various markers that constitute them, but this approach does not fully capture an overall view of their organization. Due to their

© 2026. Published by The Company of Biologists under the terms of the Creative Commons Attribution License (https://creativecommons.org/licenses/by/4.0/).

three-dimensional arrangement within the ovarian follicle, characterizing their organization using transmission electron microscopy (TEM) has been very limited until now. This v-EM study allows the authors to document the evolution of RC size and thickness during the development of germline cysts, from the germarium to stage 4, and potentially beyond. This study confirmed previous findings, namely that RC size correlates with lineage: the largest RC is formed after the first division, while the smallest is formed during the last division.

Furthermore, this work allowed a better characterisation of the membrane interdigitation surrounding the RCs. In addition, the authors highlight the important potential of v-EM for further structural analysis of the fusome, migrating border cells and the stem cell niche.

Majors comment

The output of this work can be divided into two parts. First, this work presents a technical challenge, involving image acquisition by volume electron microscopy and manual 3D reconstruction of the contours of the membranes, nuclei, RCs, and fusome in different cysts at different stages.

Secondly, this work is based on a structural study of the RCs and their associated membranes. This work is descriptive but important, although the results largely confirm previous findings, both for the structure of the RCs and their relationship to the division sequence of the cyst cells, and for the organisation of the membranes around the RCs.

Very interestingly, the authors report the spatial characterisation of membrane structures associated with and close to CRs that have already been identified (Loyer et al.). However, their characterisation is somewhat incomplete, as it lacks quantified data - how many CRs were analysed? and, above all, the characteristics of these membranes, their length and orientation according to their position and their connection in the lineage - these data could be obtained from the VEM data already collected and would be an important addition to the RC structural analysis in this work.

Following the suggestions of this reviewer, we have reduced the emphasis on the technical approach to better highlight the ring canal data. We have summarized the ring canal measurements in graphs presented in Fig. 4B, C and included the sample sizes for these measurements in the figure legend.

To gain further insight into the membrane interdigitations, we have developed a detailed model of the oocyte and four ring canals that connect to the posterior nurse cells of the stage 4 egg chamber (Fig. 5). From this model, we see that the interdigitations are longer and more abundant that in the germarium (Fig. S5), but not as extensive as in the stage 8 egg chamber (Fig. 6). The interdigitations were not all oriented in the same direction, and we did not observe an obvious correlation between interdigitation number, orientation, and lineage. We plan to continue to explore these structures in future studies.

In line with this, the authors importantly report the presence of an ER-like membrane structure lining the RCs. First, it would be nice to have statistics to support the observation of how many RCs..? Secondly, does this ER membrane structure vary according to the position of the RC in the cyst, are they related to the RC lineage?

We appreciate the reviewer's interest in this novel ER-like structure lining the ring canals. We have generated a detailed model of these structures within the stage 4 egg chamber (Fig. 5D,E). However, because we do not have data from a large number of egg chambers, we believe that performing statistics would not be appropriate.

The addition of graphs showing the quantitative data with statistics in the figures would improve understanding of the results. This is particularly the case for the characterisation of RCs according to the stage of cyst development, as shown in Figure 3. This also applies to the characterisation of RCs within a cyst and the relationship between RC size and lineage, as shown in Figure 4, and to the characterisation (thickness) of the inner part of the RC.

© 2026. Published by The Company of Biologists under the terms of the Creative Commons Attribution License (https://creativecommons.org/licenses/by/4.0/).

We have included graphs of ring canal diameter based on stage (Fig. 4B) or lineage (Fig. 4C); however, because we only have data from a few germline cysts, we have not performed any statistical analysis.

The part on the structural analysis of the fusome is interesting but still secondary to the characterisation of the RCs. This part should be moved to the results and figures after the various parts concerning the RCs.

We have deemphasized the fusome structural analysis in the results section; however, we chose to leave these images in the figures, since there could be a connection between the novel ER-like structures and the fusome.

Minor comments

The distribution of the fusome in Figure 2 is difficult to see with Hts labelling and does not really correspond to the schematic, especially in regions 2a and 2B.

We have modified the images and the schematic.

In panel C of Figure 2, it is a little disturbing that the legend is directly on the image of RC. It hides some information about the images and could be placed at the bottom of the panel. This also the case for the panel G.

We understand the possible confusion and have changed the layout in the figure.

With figure 3B, it would be good to highlight the position of cyst.

We have pseudocolored the portion that corresponds to the relevant cyst in the same color used for the reconstruction (which is now Fig. 3A).

Reviewer #1 (Significance (Required)):

As mentioned above, this work can be divided into two parts. The part corresponding to the acquisition of images by volume electron microscopy and manual 3D reconstruction is new and a great source of valuable information. The part related to the spatial characterisation of the RC is important, but corresponds more to an extension and reinforcement of previously available information than to the contribution of significant new insights. I think it will be of great interest to an audience interested in Drosophila oogenesis.

Reviewer #2 (Evidence, reproducibility and clarity (Required)):

This study presents a high-resolution volumetric analysis of germline ring canals (RCs) during Drosophila oogenesis. By combining two complementary electron microscopy techniques-Focused Ion Beam Scanning Electron Microscopy (FIB-SEM) and Array Tomography Scanning Electron Microscopy (AT-SEM)-the authors compare RC structural features at different developmental stages, ranging from the relatively small germarium to the significantly larger, later-stage egg chambers.

At early stages of oogenesis, FIB-SEM analysis confirms that the average RC size increases progressively with cyst development, in agreement with previous studies. The authors further show that lineage reliably predicts RC size (an observation previously reported, but here identified at an earlier stage in region 2a) and, importantly, that the thickness of the actin rim can also be predicted by lineage (reported here for the first time, at stage 1). FIB-SEM analysis also enables a clear delineation of the fusome, allowing for detailed characterization of its assembly and disassembly. Notably, the authors report, for the first time, structural evidence of ER-like membranes capping the inner rim of actin RCs.

At later developmental stages, AT-SEM analysis reveals that the microvilli observed by FIB-SEM evolve into extensive interdigitations extending beyond the outer rim in mid-stage egg

© 2026. Published by The Company of Biologists under the terms of the Creative Commons Attribution License (https://creativecommons.org/licenses/by/4.0/).

chambers, a structural feature detected earlier than previously reported. Moreover, by analyzing a sample in which tissue organization was disrupted during preparation, the authors demonstrate that these interdigitations preferentially occur in proximity to the RC. In addition to RC analysis at later stages, the authors use AT-SEM to readily identify small cell populations, such as the germline stem cell niche and border cells, and provide high-resolution volumetric EM data for these structures.

MAJOR COMMENT

My main comment is that we don't learn much new about the biology of these ring canals. The results primarily confirm findings from previous studies using conventional electron microscopy.

Although TEM data has been used to perform foundational studies in the field, there are limitations to this approach. Due to the size of the ring canals, it is challenging to locate them within the large volume of the egg chamber (especially at later stages). Even if ring canals can be located, they are typically not oriented the same way, so a single section is not sufficient. Although some of the results shown by our complementary vEM approaches do confirm results that have been previously reported by TEM or fluorescence microscopy, our approach provides important additional insight into structures that have been studied for many decades that would not be possible using other approaches. Further, this approach has identified a novel membrane structure lining the ring canals, and it has provided structural details of the membrane interdigitations that would not be possible with conventional electron microscopy. Further, this complementary set of vEM approaches would be applicable to the study of many other structures within other tissue types.

One particularly interesting biological question, which is briefly mentioned in the text, is whether the oocyte is the cell that inherits the majority of the fusome. Since the authors are able to reconstruct the fusome using their data, they could measure the fusome volume in each cell (especially in the two pro-oocytes) and investigate whether the cell with the larger fusome ultimately becomes the oocyte. This question has been discussed for some time, and recent studies have proposed opposing models based on fusome volume to explain how the oocyte is selected among the 16 sister cells (Nashchekin et al., Science, 2021; Barr et al., Genetics, 2024).

We appreciate the reviewer's interest in the fusome, and we agree that our approach has provided significant insight into its three dimensional structure. The rendering of the fusome was performed using a large number of small isosurface volumes, and it is therefore difficult to accurately determine the fusome volume, since additional (non-fusome) material could be included in the model. Further, the fusomes that were rendered were within the germline clusters from region 2b, where the fusome has already started to break down, so these would not provide an accurate quantification of the full fusome volume. Because the focus of the manuscript is on the germline ring canals and associated structures such as the interdigitations (which we have tried to further streamline in this revised version), we believe that additional analysis of the fusome is outside of the scope of this work.

MINOR COMMENT

• The fluorescent markers used in the fly stocks are neither described in the Materials and Methods section nor depicted in the figures.

We apologize if this was not clear in the original manuscript. Based on the comment from Reviewer #3 (see below), we have repeated the Hts staining using flies that do not have CheerioYFP in the background. We have also clarified the materials and methods section to indicate the panels that correspond with each strain used.

• The authors should quote (Nashchekin et al., Science, 2021) when mentioning unequal partionning of the fusome (p4) and oocyte determination (p12).

We have added the reference to these parts of the manuscript.

© 2026. Published by The Company of Biologists under the terms of the Creative Commons Attribution License (https://creativecommons.org/licenses/by/4.0/).

• P11-12, when mentioning electron dense regions reflecting strong cell-cell adhesion, the authors could refer to (Fichelson et al. Development, 2010), where AJ have been described around ring canals.

We have added the reference to this part of the manuscript.

• Figure 2A: The schematic diagram (4th line) is not explained in the figure legend.

We have updated the figure legend to describe this schematic.

• Figure 2D: Please clarify whether the RC stage shown corresponds to stage 1 or stage 10, as indicated in panel 2E. Alternatively, are these examples representing the minimum and maximum RC sizes observed across the entire dataset?.

These were not meant to be examples of the minimum and maximum ring canal sizes observed across the dataset. Instead, they were used to demonstrate the significant expansion that occurs during oogenesis. In the updated version of this figure, this panel has been removed.

• Figure 5D: Please specify which panel in 5B this corresponds to.

• Figure 5E: Please specify which panels in 5B this corresponds to. The two green boxes are not defined. Why is there a grey background under the ovariole assembly?

• Figures 5G, 5H: Does panel 5G correspond to the left green box in 5E, and 5H to the right green box in 5E? Please clarify.

We have modified Figure 5 and merged it with the figure 6. In this updated format, panels 5B and 5E have been removed.

• Figure 6: The figure title is not on the same page as the figure itself.
We have made this change.

• Figure 6A: The black box marking the germarium is not defined.
In this revised version, we have modified Fig. 6, and this panel has been removed.

• Figure 6B-E: The arrows point to long interdigitations. However, arrowheads (which are not mentioned in the legend) appear to indicate the RC outer rim. Please specify this clearly in the figure legend.
In the updated version of Fig. 6, these arrowheads have been removed.

Reviewer #2 (Significance (Required)):

I am not an expert in electron microscopy, so I cannot comment in detail on these techniques, but they appear to bridge the gap between conventional EM and optical microscopy in terms of resolution, user-friendliness, and other aspects. This is technically interesting, although these EM approaches have been previously described and applied. The images and movies are beautiful and clearly presented.

My main comment is that we don't learn much new about the biology of these ring canals. The results primarily confirm findings from previous studies using conventional electron microscopy.

One particularly interesting biological question, which is briefly mentioned in the text, is whether the oocyte is the cell that inherits the majority of the fusome. Since the authors are able to reconstruct the fusome using their data, they could measure the fusome volume in each cell (especially in the two pro-oocytes) and investigate whether the cell with the larger fusome ultimately becomes the oocyte. This question has been discussed for some time, and recent studies have proposed opposing models based on fusome volume to explain how the oocyte is selected among the 16 sister cells (Nashchekin et al., Science, 2021; Barr et al., Genetics, 2024).

© 2026. Published by The Company of Biologists under the terms of the Creative Commons Attribution License (https://creativecommons.org/licenses/by/4.0/).

Reviewer #3 (Evidence, reproducibility and clarity (Required)):

Kolotuev et al. used two volume-based electron microscopy based approaches to identify, segment, and document the changes in intercellular bridges, or ring canals, in early egg chambers of the fruit fly, Drosophila melanogaster. Using array tomography and focused ion beam scanning electron microscopy, Kolotuev et al., provide a high resolution and content rich lineage analysis of ring canal size, shape and orientation among early and late egg chambers. Their analysis included parameters such as the presence and shape of the fusome, the recruitment of actin to the inner ring, and development of membrane fingers that presumably spatially stabilize such structures. Last, Kolotuev and co-authors highlight additional aspects of their dataset including a reconstruction of the border cell cluster in stage 9 egg chambers. The data presented are a treasure trove of the ultrastructural features of the developing dipteran germline and subsequent ovarian follicle development. The data presented represent the highest resolution 3D dataset available and thus are a valuable worthwhile contribution to the field. My overall impression is that this paper sits intellectually between a valuable method and a loose experimental manuscript. This critique is not requesting additional experimental evidence because the data are unique and are the foundation for a new experimental paradigm. But there is not sufficient detail presented to be a full method, nor any hypothesis testing to be considered experimental. I suggest the authors consider amplifying their methods in detail and then note that using these methods provide a foundation for additional future investigations (as mentioned in the discussion). Problems with data interpretation and presentation should be addressed before publication. Below are the major and minor concerns that I believe need to be considered.

Major comments:

In general images in figures are thought provoking, however changes to figure layout and design should be considered to better highlight the results. For instance, I don't know how to follow figure 1a. The arrow leads from a whole ovary to an ovulated egg with an ovariole strand connecting the two. What is the purpose of the arrow? Is it to represent time? And why is the mature egg in the figure when no data regarding this stage is presented. The authors should consider removing the mature egg and helping the reader understand that the ovariole is a subset of the whole ovary. They might do this by putting a box around a single ovarile in the whole ovary to indicate their ovariole illustration. Several other figures have similar problems. Throughout the authors used black and white arrows on black and white EM data and these arrows were lost. Color should be considered to effectively point out what they want the reader to see.

We have modified the layout of Fig. 1 and added additional explanation to the introduction and figure legend to guide readers through the introduction to the system. We have also added color to some of the arrows throughout the manuscript.

Can the authors provide additional information for the genotypes used? For instance the Cherrio-YFP (which might affect actin). When what this used and can the authors provide information on how this affected the data between when it was used and when it was not used. Additionally, why was analysis done in transgenic flies over fully wild-type?

We have repeated the Hts staining in Fig. 2A in flies that do not express Cheerio-YFP and have made the appropriate changes to the methods section. For the AT-SEM experiment, we chose to use this genetic background since it would align with that of the negative controls that we often use in RNAi or over-expression experiments. FIB-SEM datasets were collected while imaging other tissues of the fly, so the choice of that genotype was not intentional. However, these datasets provided us with the opportunity to do this proof-of-concept work without such a large financial investment in the acquisition of new image stacks. In the future, we hope to expand this work to generate additional datasets from flies of different genotypes.

Figure 1 seeks to lay out the ovary system and narrow the reader into the stages that will be analyzed in subsequent figures. Figure 1B is meant to show the types and kinds of electron microscopy, however lacks a full detailed description and legend for each of the colored arrows. And to that fact, so does figure S1. The authors need to provide additional information so the

© 2026. Published by The Company of Biologists under the terms of the Creative Commons Attribution License (https://creativecommons.org/licenses/by/4.0/).

reader can glean what the authors point they are trying to convey. In addition, the authors might add pros and cons to each. I know this was attempted in S1, but did not fully come across.

We appreciate this feedback, and we have modified the layout of Figure 1 and updated Figures S1 to better highlight the technical challenge of EM in general and benefits of vEM in particular.

Figure 1 and 2 seek to set up both the biological and technical system to be understood. The authors might consider combining the two figures and eliminate elements that don't represent a result of any kind (Figure 1B, 2B, 3D and 3F). Or more fully explain the result and point they are trying to make with these illustrations. I fully understand and appreciate what they are trying to get across, but it does not come across clearly. For example, I don't know how figure 2B effectively gets across the point that rotation of the image has an effect on how it is sliced and segmented in EM data. Not sure it is necessary. Furthermore, what is the bottom panel with a green ring canal supposed to allow us to interpret or conclude? The same for 3D and F. The result in 3E is far more interesting and should be two panels that emphasize the growth characteristics between young and old rings or those of M1 and M4.

We greatly appreciate these suggestions, and we have modified and reorganized several figures to make the flow of scientific ideas easier to follow. We have moved panel 1B to the supplementary figure and gave additional indications in the text as to the differences between the EM methods. We have moved panel 2B to the supplementary material. We have moved Fig. 3D to Fig. S5A,B. Fig. 5 now provides more extensive rendering of membrane interdigitations from the stage 4 egg chamber. We have chosen to leave Fig. 3F to allow readers to compare the novel ER-like structures within the ring canals to the fusome that is present within younger germline clusters.

The HTS and actin stain in figure 2A overlap significantly and obscure the fusome staining. Can the authors confirm that there is no bleed through in their staining and imaging procedure?

We have repeated this staining and can confirm that there was no bleed through between the two channels.

The data in Figure 2C are critical to showing the z-resolution enhancement of sectioned EM. However, the use of green psuedocolor only in one panel is confusing. Can the authors duplicate the whole panel and provide one without and one with psuedocolor? This would be ideal for fully orienting the reader to the sectioning and setting them up to understand the rest of the figures.

In the revised version of Figure 2, we have split the sections into two rows of panels; we have added the pseudocolor to every other section (in the bottom row of panels).

The results section for figure 2 does outline the results presented. For example, the germarium contains syncytia of differing stages and ring canals with intervening fusomes... It does more to talk about the pros and cons of different technical aspects and their difficulty This should be saved for the rationale or the discussion. Rather the section should outline the results presented.

We have modified the layout of figure 2 in order to describe the system in a more straightforward manner with a smoother transition from Figure 1 while further explaining technical points.

I appreciate the color coding of the differentially segment cysts in Figure 3. The color coding helped orient me to which cysts were being evaluated. However I found the lack of detail bothersome. For instance, which ring canals are in the two panels of D? Are they M1 or M4?

With the additional analysis of the interdigitations in the stage 4 cluster, we have moved panel D to Fig. S5. We did not have enough coverage of the region 2a cluster (red) to determine lineage, but we have added a statement to the legend to indicate that the ring canal shown in Fig. S5B is an M1 ring canal.

© 2026. Published by The Company of Biologists under the terms of the Creative Commons Attribution License (https://creativecommons.org/licenses/by/4.0/).

Also, the presentation of ring canal size and distribution should be presented in a graph. Statistics are not necessary, but a dot-plot would go a long way to presenting the result. Two plots can add value, one in which the ring canals for each phase is shown, and the other is the distribution of sizes for each cyst.

We have added these graphs in Fig. 4B, C.

Lastly, the results section for figure 3 interprets the membrane bound vesicles in the ring canal as "ER-like". This should be removed since they neither look ER-like to me, nor have been shown to be ER in the data.

We appreciate this suggestion, and although we cannot be absolutely certain of the identity of these structures without further study, with our additional analysis of the stage 4 egg chamber, we are further convinced of the similar appearance of these novel structures and the ER in other regions of the nurse cell (Fig. 5). We have clarified this point in the text.

Figure 4A is not called out specifically in the results and thus should be interpreted or removed from the figure.

In this revised version, we have removed panel 4A.

Figure 5 was confusing. I understand the authors wanted to show the wafer and the ribbons, however, this is not a result and does not offer any interpretation of a result and is thus confusing on why it is in the figure. If this were a method paper, I would understand its presence.

We have removed this panel from the figure.

Can the authors comment on the shape of the nuclei in older egg chambers? They are not round at all. I am interested in whether this is a fixation artifact or the real ultrastructure of the nuclei. Of the border cell nuclei for instance. If it is an artifact, this should be added to the discussion.

Some of the nuclei appear to have a peculiar shape in the cross-section. We cannot entirely exclude the role of the fixation in the shape irregularities. However, since not all the nuclei are subject to this phenomenon, we are inclined to attribute it to the intrinsic qualities of the late-stage nuclei. In numerous cases, different tissue and cell stages determine the shape of the nucleus, which frequently deviates from a spherical shape.

Although data from "imperfect" samples is interesting, consider relegating Figure 6 to the supplement section, as it takes away from the pre-existing narrative flow established in the paper.

In this draft, we have combined parts of figures 5 and 6, and much of the data from the imperfect sample has been removed.

Interpretation of the data throughout the results should be left to the discussion section. For instance, interpretation of Figure 4 results on page 14 beginning with "these data demonstrate the importance...". The importance is not related to the result, but rather discussion of past and future studies.

We have removed this sentence from the results.

In another example, Figure 5I is introduced and discussed in the results section on page 15, second whole paragraph with an overall introduction/discussion on junctions, which convolutes the actual result. Discussion of future studies or how structures like the novel membrane fingers should be viewed in a larger biological context, should not be in the results.

We have made this change.

<u>Minor comments:</u>

Remove words such as "pseudo-timelapse", they invoke precision on a point that is imprecise.

© 2026. Published by The Company of Biologists under the terms of the Creative Commons Attribution License (https://creativecommons.org/licenses/by/4.0/).

This has been removed.

Re-consider the acronyms for ring canal and egg chamber.

We have removed these acronyms.

Consider finding another way to call out each supplemental movie other than with another acronym.

We have added small icons to indicate that a supplemental movie is associated with a given figure or panel.

Reviewer #3 (Significance (Required)):

The present manuscript is a technical advance in the field. The use of serial EM imaging with two separate modalities, on what is considered to be a challenging problem in the field, represents a useful technical advance. Light microscopy has thus far limited the resolution to which we can understand the spatial organization and the cellular features there in that regulate germline development. This manuscript brings to bear two serial EM methods to begin approaching this problem. The audience for this work are those working at the forefront of understanding germline architecture and development. I make these statements as an expert in live and super resolution of fruit fly egg chamber development, in addition to having performed 3D SEM in past works.

Original submission

First decision letter

MS ID#: jcs.264713

MS TITLE: Complementary Volume Electron Microscopy-based approaches reveal ultrastructural changes in germline intercellular bridges of D. melanogaster

AUTHORS: Irina Kolotuev; Sophie Khazanov; Abigayle Williams; Caroline Kizilyaprak; Stephanie Pellegrino; Lindsay Lewellyn

ARTICLE TYPE: Review Commons Transfer

Dear Dr Lewellyn,

We have now reached a decision on the above manuscript.

As you will see, the reviewers gave favourable reports but raised some critical points that will require amendments to your manuscript. I hope that you will be able to carry these out because I would like to be able to accept your paper, depending on further comments from reviewers.

We have reviewed a previous version of this manuscript, and the revised version has not changed substantially. The main novelty lies in the additional insight into membrane interdigitations, made possible by a detailed 3D model of the oocyte and the four ring canals connecting it to the posterior nurse cells in the stage 4 egg chamber (Fig. 5). The authors have also added new graphs summarizing ring canal diameter measurements (Fig. 4).
Major comment:
As previously noted by several reviewers, this study remains purely descriptive and does not significantly advance our understanding of Drosophila oogenesis, ring canals, or fusome biology. To enhance the scientific impact of the study, we previously suggested that the authors quantify the fusome volume in each of the two pro-oocytes. Asymmetry in fusome content between the two

© 2026. Published by The Company of Biologists under the terms of the Creative Commons Attribution License (https://creativecommons.org/licenses/by/4.0/).

pro-oocytes has been linked to oocyte fate specification and represents an important question in the field.
In response to our comments, the authors state that accurate determination of fusome volume is complicated by the difficulty of disentangling fusomal material from additional, non-fusomal volumes. Consequently, they did not pursue further experiments, arguing that a detailed analysis of the fusome lies beyond the scope of the present study. However, quantification of the external "envelope" of the fusome has previously been performed using various EM techniques and fluorescent markers. This analysis should be feasible using the authors' own methods and would allow a meaningful contribution to the ongoing debate. Notably, in Fig. 3D, the authors already present high-quality images of the fusome obtained with their approach.
Aside from these points, the revisions are limited to improved figure readability, rephrasing of the Results section, an expanded Discussion, and the addition of relevant references.
In its current form, we agree with the other reviewers that this study "is positioned between a valuable methodological contribution and an insufficiently developed experimental manuscript."
Minor Comments
1) Figure 2A: It would be more consistent to represent one or two germ cells larger in the schematic cartoon, as observed in the EM image below.
2) Figure 2C: "Fluorescence" is misspelled as "fluoreSence."
3) Figure 5G: Please provide a more detailed description.

First revision

<u>Author response to reviewers' comments</u>

<u>**Response to Reviewers**</u>

We greatly appreciate the additional feedback provided by the three reviewers. We have responded to each of their comments below.

Reviewer 1: We have reviewed a previous version of this manuscript, and the revised version has not changed substantially. The main novelty lies in the additional insight into membrane interdigitations, made possible by a detailed 3D model of the oocyte and the four ring canals connecting it to the posterior nurse cells in the stage 4 egg chamber (Fig. 5). The authors have also added new graphs summarizing ring canal diameter measurements (Fig. 4).

<u>Major comment:</u>

As previously noted by several reviewers, this study remains purely descriptive and does not significantly advance our understanding of Drosophila oogenesis, ring canals, or fusome biology. To enhance the scientific impact of the study, we previously suggested that the authors quantify the fusome volume in each of the two pro-oocytes. Asymmetry in fusome content between the two pro-oocytes has been linked to oocyte fate specification and represents an important question in the field. In response to our comments, the authors state that accurate determination of fusome volume is complicated by the difficulty of disentangling fusomal material from additional, non-fusomal volumes. Consequently, they did not pursue further experiments, arguing that a detailed analysis of the fusome lies beyond the scope of the present study. However, quantification of the external "envelope" of the fusome has previously been performed using various EM techniques and fluorescent markers. This analysis should be feasible using the authors' own methods and would allow a meaningful contribution to the ongoing debate. Notably, in Fig. 3D, the authors already present high-quality images of the fusome obtained with their approach.

We still believe that that a detailed analysis of the fusome is beyond the scope of the paper and that additional electron microscopy and fluorescence experiments related to this question would not strengthen the manuscript. However, we have used our model of the fusome to provide a rough estimate of the volume of fusome material within each of the two pro-oocytes from the two germline clusters shown below.

© 2026. Published by The Company of Biologists under the terms of the Creative Commons Attribution License (https://creativecommons.org/licenses/by/4.0/).

We would like to provide the following caveats regarding these data, and based on these limitations, we do not want them included in the final manuscript. However, we are happy to provide this information to the reviewer. (1) The isosurface tool was used to render the fusome material within each of the two pro-oocytes. This tool does not allow us to distinguish between fusome and non-fusome material. (2) Some of the isosurface objects captured not only the fusome but also the ring canals; when possible, we subtracted the volume of the included ring canal. (3) To capture as much of the fusome as possible, some isosurface objects overlapped each other, so some fusome material might have been captured within multiple objects. (4) Because of the position of the fusome in some areas of the germline, and the nature of the isosurface tool, some fusome material might not have been captured in our model. With these caveats in mind, we provide the following quantification of fusome material in the two pro-oocytes from the two germline clusters shown in Fig. 3.

NOTE: We have removed unpublished data that had been provided for the referees in confidence.

Aside from these points, the revisions are limited to improved figure readability, rephrasing of the Results section, an expanded Discussion, and the addition of relevant references. In its current form, we agree with the other reviewers that this study "is positioned between a valuable methodological contribution and an insufficiently developed experimental manuscript."

<u>Minor Comments</u>

1) Figure 2A: It would be more consistent to represent one or two germ cells larger in the schematic cartoon, as observed in the EM image below.

We have modified the cartoon in Fig. 2A to reduce the number of germline cell clusters that are shown.

2) Figure 2C: "Fluorescence" is misspelled as "fluoreSence."

We have corrected this and appreciate the reviewer catching this mistake.

3) Figure 5G: Please provide a more detailed description.

We have added additional detail to the legend for Fig. 5.

Reviewer 2: This manuscript delivers an unprecedented EM-resolution view of intercellular bridges in Drosophila ovaries called ring canals. It offers insight into their ultrastructural configuration and highlights new features not seen before. It is also a technical advance in EM preparation of this tissue.

SUGGESTIONS TO AUTHORS

<u>Minor Comments</u>

1) All my comments have been satisfied, however, authors should consider adding arrow to Fig. 5G. This is minor and does not need re-review.

Arrows have been added to Fig. 5G to highlight two different interdigitations that extend in different directions from the same cell-cell contact.

Reviewer 3: The possibility of observing 3D cellular organisation in tissues at nanometre resolution is a hope for many cell biologists. Here, the authors have combined two volume electron microscopy approaches with scanning electron microscopy: Focused Ion Beam (FIB-SEM) and Array Tomography (AT-SEM) to study the evolution of the shape and organisation of cytoplasmic bridges, the 'ring canals' (RCs) in the Drosophila ovarian follicle that connect nurse cells and oocyte. This type of cytoplasmic link, found in insects and humans, is essential for oocyte development. RCs

© 2026. Published by The Company of Biologists under the terms of the Creative Commons Attribution License (https://creativecommons.org/licenses/by/4.0/).

have mainly been studied using light microscopy with various markers that constitute them, but this approach does not fully capture an overall view of their organization. Due to their three-dimensional arrangement within the ovarian follicle, characterizing their organization using transmission electron microscopy (TEM) has been very limited until now. This v-EM study allows the authors to document the evolution of RC size and thickness during the development of germline cysts, from the germarium to stage 4, and potentially beyond. This study confirmed previous findings, namely that RC size correlates with lineage: the largest RC is formed after the first division, while the smallest is formed during the last division. Furthermore, this work allowed a better characterisation of the membrane interdigitation surrounding the RCs. In addition, the authors highlight the important potential of v-EM for further structural analysis of the fusome, migrating border cells and the stem cell niche.

SUGGESTIONS TO AUTHORS

In this revised manuscript, Irina Koltuev and her colleagues have improved their manuscript. They have address most of the points raised by the reviewers. The authors have modified the figures by integrating new image analysis and quantifications. They have also refined the presentation to highlight their significance. The manuscript has improved since the last submission and I recommend its publication in Journal of cell Science.

Minor point

1) Line 19 refers to Supplementary Movie 4, which is not included in the figure legends.

We have removed this reference to Supplementary Movie 4 and appreciate the reviewer catching this mistake.

Second decision letter

MS ID#: jcs.264713R1

MS Title: Complementary Volume Electron Microscopy-based approaches reveal ultrastructural changes in germline intercellular bridges

Authors: Irina Kolotuev; Sophie Khazanov; Abigayle Williams; Caroline Kizilyaprak; Stephanie Pellegrino; Lindsay Lewellyn
Article Type: Review Commons Transfer

Dear Dr Lewellyn,

I am happy to tell you that your manuscript has been accepted for publication in Journal of Cell Science, pending standard publication integrity checks.

Thank you for sending your manuscript to Journal of Cell Science through Review Commons.

© 2026. Published by The Company of Biologists under the terms of the Creative Commons Attribution License (https://creativecommons.org/licenses/by/4.0/).
